# MUC2 expression modulates immune infiltration in colorectal cancer

**DOI:** 10.3389/fimmu.2024.1500374

**Published:** 2025-01-24

**Authors:** Christophe M. Raynaud, Ayesha Jabeen, Eiman I. Ahmed, Satanay Hubrack, Apryl Sanchez, Shimaa Sherif, Ahmad A. Al-Shaibi, Bernice Lo, Jessica Roelands, Davide Bedognetti, Wouter Hendrickx

**Affiliations:** ^1^ Tumor Biology and Immunology Laboratory, Research Branch, Sidra Medicine, Doha, Qatar; ^2^ Department of Biomedical Science, College of Health Sciences, Qatar University, Doha, Qatar; ^3^ Laboratory of immunoregulation, Research Branch, Sidra Medicine, Doha, Qatar; ^4^ College of Health and Life Sciences (CHLS), Hamad bin Khalifa University (HBKU), Doha, Qatar; ^5^ Department of Pathology, Leiden University Medical Center, Leiden, Netherlands; ^6^ Clinical and Experimental Oncology and Hematology, Ospedale Policlinico San Martino, Genova, Italy

**Keywords:** mucins, MUC2, immune infiltration, spheroids, colon cancer

## Abstract

**Introduction:**

Colorectal cancer (CRC) is a prevalent malignancy with significant morbidity and mortality worldwide. A deeper understanding of the interaction of cancer cells with other cells in the tumor microenvironment is crucial to devise effective therapeutic strategies. MUC2, a major component of the protective mucus layer in the gastrointestinal tract, has been implicated in CRC progression and immune response regulation.

**Method:**

In this study, we sought to elucidate the relationship between MUC2 expression and immune infiltration within CRC using *in vitro* models involving two well-established cell lines, HT-29 and LS-174T. By employing CRISPR-mediated MUC2 knockout, we investigated the influence of MUC2 on tumor immune infiltration and its interplay with T cells and NK cells enriched peripheral blood mononuclear cells (PBMCs) in 3D spheroid cultures.

**Results:**

While MUC2 was more abundant in LS-174T cell line compared to HT-29, its knockout resulted in increased immune infiltration solely in the HT-29 cell line, but not in the LS-174T cell line. We revealed that the removal of MUC2 protein was compensated in LS-174T by the expression of other gel-forming mucin proteins (MUC6, MUC5B) commonly expressed in the gastrointestinal epithelium, while this was not observed in HT-29 cell line.

**Conclusion:**

Our study is the first to demonstrate that MUC2 functions as a physical barrier to immune infiltration in colorectal cancer (CRC) *in vitro*. In HT-29 cells, MUC2 knockout increased immune infiltration, while in LS-174T cells, compensatory expression of other mucins (MUC6, MUC5B) maintained the barrier. These findings reveal the complexity of mucin biology in CRC and suggest that targeting mucin pathways could be a novel therapeutic approach.

## Introduction

Colorectal cancer (CRC) is a complex disease influenced by various genetic, epigenetic, and environmental factors (1–3). While significant advancements have been made in understanding CRC biology, the role of the tumor microenvironment and its interactions with tumor cells remains an area of active investigation. The tumor microenvironment plays a critical role in modulating tumor growth, invasion, and immune surveillance, ultimately determining the overall clinical outcome of CRC patients (1).

Mucins play an important role in the colon as it is the primary component of the protective mucus layer lining the colon’s surface. This mucus layer acts as a barrier against harmful substances, bacteria, and pathogens, preventing them from directly interacting with the colon’s epithelial cells (4). Additionally, mucin aids in lubricating and facilitating proper bowel function. MUC2 is an essential member of the mucin family in the colon as it is a gel-forming mucin along with MUC5AC, MUC5B, MUC6, and MC19 (5). Alterations in MUC2 expression have been implicated in various gastrointestinal disorders and malignancies, including colorectal cancer (CRC) (6). Notably, changes in MUC2 expression have been associated with CRC progression and prognosis. Patients with low MUC2 expression in CRC tumors have a significantly poorer overall survival compared to patients with a high MUC2 expression. This highlights the potential of MUC2 as a prognostic biomarker (7). Nevertheless, the impact of MUC2, the aberrant glycosylation of mucins, and their interaction with the immune system in CRC are active areas of investigation.

In the context of CRC subtypes, mucinous CRCs are characterized by elevated MUC2 expression compared to normal colon tissue, serving as a hallmark feature (8–10). Conversely, non-mucinous CRCs typically exhibit a lower MUC2 expression compared to mucinous CRC (8). The loss of MUC2 expression in CRC has been linked to increased proliferation of intestinal epithelial cells in response to mucosal inflammation (11).

Multiple factors regulate MUC2 expression in colonic epithelial cells. Studies have shown that methylation of the MUC2 gene promoter is significantly lower in mucinous CRC lines compared to non-mucinous lines, correlating with higher mucin protein expression in mucinous CRCs (12). The tumor suppressor protein p53 is another crucial regulator of MUC2 expression; loss of functional p53 leads to the downregulation of MUC2 (13). This transcriptional upregulation of MUC2 by p53 has been observed in various cell lines (14), consistent with the reduced incidence of p53 mutations in mucinous carcinomas (13). In contrast, non-mucinous CRCs often display high rates of p53 mutation and low MUC2 expression (14). Moreover, the mitogen-activated protein kinase (MAPK) signaling pathway has been identified as another regulator of MUC2 gene transcription (15). Notably, MAPK signaling is upregulated in CRCs harboring KRAS mutations and in the context of chronic inflammation, both of which are common features of mucinous CRC (16).

In summary, MUC2’s critical role in the gastrointestinal tract, its association with CRC progression, and its regulation by various factors underscore the importance of investigating its impact on the immune landscape within the tumor microenvironment. We previously found that, overall, mucinous colon tumors are characterized by a reduced Th1-oriented immune response as determined by transcriptomic analysis using the immunologic constant of rejection (ICR) (1). Considering the described barrier function of the mucus layer, we proposed that infiltration of immune cells into the tumor might be hampered in mucinous cancers. Therefore, understanding these intricate relationships may pave the way for novel therapeutic approaches targeting MUC2 and its interactions with the immune system to improve outcomes for CRC patients.

In this study, we aimed to investigate the role of MUC2 in CRC immune infiltration, focusing on the interaction between MUC2-altered CRC cells and T cells and NK cells. We utilized CRISPR-mediated gene editing techniques to specifically knockout MUC2 expression in HT-29 and LS-174T cell lines, two widely studied CRC models. These cell lines provided an ideal platform to assess the impact of MUC2 modulation on immune cell infiltration into the tumor and its interplay with immune cells in a controlled and reproducible *in vitro* setting. Additionally, to better recapitulate the tumor microenvironment, we employed 3D spheroid cultures, a well-established model that more closely mimics the cellular architecture and interactions present in solid tumors than a traditional monolayer. By appending T-cell- and NK-cell-enriched PBMCs into the 3D spheroid cultures, we aimed to simulate the immune environment within the CRC microenvironment.

## Materials and methods

### Cell lines and culture

LS-174T (CL-188), HT-29 (HTB-38), and LoVo (CCL-229) cell lines were purchased from ATCC. Original and modified cell lines were cultured in advanced RPMI (Gibco, #12633012) complemented with 10% FBS (Sigma, #F4135-500ML, RRID: SCR_008988), Glutamax (Gibco, #35050061), and antibiotic–antimycotic (Gibco, #15240096) (complete media). The cells were cultured at 37°C, 5% CO_2_, and 95% humidity. The cells were detached using TrypLE express enzyme (Gibco, #12605036). For 2D culture, T-75 (Falcon, #353136) or 96-well plates (Falcon, #353072) were used. For 3D culture, low-adherence Nunclon Sphera U-bottomed 96-well plates were used (Thermo, #174925).

### RNA extraction

After culture and detachment, the cells were rinsed in DPBS (Gibco, #10010-031), and dry cell pellet was then used for RNA extraction as follows. RNA was extracted using Maxwell^®^ RSC simplyRNA Cells Kit (Promega, #AS1390) according to the manufacturer’s recommendations using Maxwell^®^ RSC Instrument (Promega, #AS4500). RNA was recovered with 50 µL of RNase free water and measured with QuantiFluor^®^ RNA System (Promega, #E3310). The RNA was stored at -80°C until use.

### Q-PCR

One microgram of RNA was used for reverse transcription using TaqMan™ Reverse Transcription Reagents (Invitrogen, #N8080234) using random hexamer following the manufacturer’s recommendation. cDNA was diluted 20 times with DNA/RNA free water. TaqMan™ Gene Expression Master Mix (Applied biosciences, #4369016) was used together with Hs03003631_g1 (for Eukaryotic 18S rRNA) and Hs03005103_g1 (for MUC2) TaqMan probes (Thermo Fisher Scientific, #4331182) according to the manufacturer’s recommendation. Real-time PCR was run in 96-well plates on QuantStudio 12K flex system (Thermo Fisher Scientific). Q-PCR was done in triplicate for each sample, and data were analyzed by gene expression comparison using ΔΔCT on (QuantStudio 12K Flex Realtime PCR system software V1.2.2).

### Western blot

After culture, 5 × 10^6^ cells of cells for 2D culture or 30 spheroids at day 5 for 3D culture were harvested and lysed in lysis buffer containing 10 mmol/L HEPES (Sigma Aldrich Co., #H0887-20ML), 150 mmol/L NaCl (Sigma Aldrich Co., #S9888-1KG), 1 mmol/L ethylene glycol-bis(β-aminoethyl ether)-*N,N,N′,N′*-tetraacetic acid (Merck, #324626-25GM), 0.1 mmol/L MgCl_2_ (Sigma Aldrich Co., #M8266-100G), 0.5% Triton X-100 (IBI Scientific, #7100, pH 7.4) containing 1 mM PMSF (Merck, #10837091001), and 10 mM N-ethyl maleimide (NEM) (Merck, #360768-37-2). Moreover, 50 mg lysate as input for each condition was loaded onto 4%–15% TGX gels (Bio-Rad, #4561084). The proteins were then transferred onto nitrocellulose membrane (Bio-Rad, #1704270) and blotted with antibodies against the proteins of interest. The Spectra™ Multicolor High Range Protein Ladder (Thermo Fisher Scientific, #26625) was used to assess the molecular weight of the detected proteins. MUC2 antibody (CCP58) (Novus, #NBP2-25221), HRP-conjugated β actin monoclonal antibody (Proteintech, #HRP-60008), mouse anti-hMuc5AC antibody (Thermofisher, #MA5-12178), and anti-calnexin antibody (Cell Signaling, #2679T) were used as primary antibody as well as anti-mouse IgG (H+L) antibody and human-serum-adsorbed and peroxidase-labeled (Seracare, #5450-0011) secondary antibody. The blots were revealed by adding SuperSignal West Femto HRP substrate (Thermo Scientific #34095), and the images were captured on a ChemiDoc MP imaging system (Biorad).

### CRISPR cell engineering

#### gRNA design

MUC2 gene sequence was obtained from ensembl human genome assembly GRCh38.p14 HSCHR11_3_CTG1: 149,268-178,812. IDT custom gRNA design tool was used to design gRNA along exon 2 of transcript ID (ENST00000643422.1).

Five gRNAs were designed as indicated in **Table 1**.

The location along exon2 of MUC2 of each gRNA is shown in **Supplementary Figure S1A**. Forward and reverse primers were ordered accordingly to be inserted in the appropriate plasmids as described below.

**Table 1:** Sequence of each gRNA designed for MUC2 K.O.

gRNA name	sequence
MUC2 gRNA1	CCACTACAAGACCTTCGACG
MUC2 gRNA2	CCACCTGGCTGTGCTTAACG
MUC2 gRNA3	CTTGTAGTGGAAGTTGCCCC
MUC2 gRNA4	CAGGATGGACTCCACCCCGG
MUC2 gRNA5	TTCCACTACAAGACCTTCGA

#### *In vitro* gRNA synthesis

The gRNAs were synthesized using the GeneArt™ Precision gRNA Synthesis Kit (Thermo Fisher Scientific #A29377) as per the manufacturer’s recommendation. The concentration of gRNA was determined by using the Qubit^®^ RNA BR Assay Kit (Thermo Fisher Scientific #Q10210) as per the manufacturer’s recommendation and diluted at 250 ng/µL final concentration and stored at -80C until use [3-5].

#### Cell transfection

Electroporation was performed using Neon transfection system (Life Technologies, #MPK5000) with Neon™ Transfection System 10 µL Kit (Life Technologies, #MPK1096) using 1 µg of DNA for 1 × 10^5^ cells in a 24-well plate according to the manufacturer’s recommendation. Electroporation protocol for each cell line was identified using pmaxCloningTM vector (Lonza, #VDC-1040). The optimal protocol for each cell line is indicated in **Table 2**. The transfection was performed using TrueCut™ Cas9 Protein v2 (Thermo Fisher Scientific, #A36496) along with pre-synthesized gRNA. The reaction conditions were set to 250 ng of gRNA and 1 µg of TrueCut™ Cas9 Protein v2 in a total volume of 5 µL of R buffer. Each cell line was transfected using its optimized program.

**Table 2:** Optimal electroporation setup for each cell line.

CELL LINE	VOLTAGE (V)	WIDTH (ms)	PULSES
HT-29	1700	20	1
LS-174T	1400	20	2

#### Bulk analysis

At 72 h after electroporation, half of the cells were collected and washed with DPBS and then lysed in 10 µL cell lysis buffer complemented with 0.4 µL of protein degrader from GeneArt™ Genomic Cleavage Detection Kit (Thermo Fisher Scientific, #A24372). PCR was performed directly on cell lysis using the primers provided in **Table 3**, and the relative location of primers to gRNA is provided in **Supplementary Figure S1A**, with Amplitaq gold 360 master mix (applied biosystem, #4396790) as per the manufacturer’s recommendation with annealing at 60°C and an elongation step of 30 s.

The PCR product was used for Sanger sequencing and analyzed as described below.

**Table 3:** Primers used for PCR and sanger sequencing of MUC2 exon 2.

Primer name	Sequence	Tm
PCR/seq1 MUC2 primer FW	TGTGCTGGGCTCGAAGCTGCTTC	64.1
PCR/seq1 MUC2 primer Rev	GGTGTCATCCTTGATGGTCAGCAGG	61.4
PCR/Seq2 MUC2 primer Fw	TCCACTACAAGACCTTCGACGGG	60.9
PCR/seq2 MUC2 primer Rev	CCCAAACCTCAAGGCTGTTCTCCTG	61.6

#### Sanger sequencing

The same primers used for PCR were used for sanger sequencing. Briefly, PCR products were purified using Exosap-IT (Thermo Fisher Scientific, #75001) followed by labeling using BigDye™ Terminator v3.1 Cycle Sequencing Kit (Thermo Fisher Scientific #4337455) as per the manufacturer’s recommendation. Sanger products were cleaned using DyeEx™ 96 kit (Qiagen, #63204) as per the manufacturer’s recommendation. Sanger sequences were recorded on ABI3500 sequencer (Applied biosystem, #4406016).

#### Sanger sequence analysis

Synthego ICE (https://ice.synthego.com/#/) [6] online tool was used to analyze the indels in bulk and for each individual clone (after sorting as described below). The selected clone had indel on both allele indel nonmultiple of three, inducing frame shift and therefore K.O.

#### Single cell sorting

Cells were harvested and blocked in PBS with 5% FBS and 1% BSA, and cell clumps were removed on a 40-µm cell strainer (Falcon, #382235). Single-cell suspension was analyzed and sorted on SORP FACSAriaIII (BD Biosciences Special Order Research Product). Data were processed with BD FACSDiva™ Software V8.0.1 (BD Biosciences). Doublets were excluded by FSC-W × FSC-H and SSC-W × SSC-H analysis. A dumping channel was used to eliminate auto-fluorescent cells with 405-nm violet laser and 525/50 emission filter. During cell sorting, single-cell sort mask was applied, and one cell was seeded per well of a 96-well plate in 200 µL of complete media supplemented with 10 µM of rock inhibitor (Y-27632) (Stem Cell Technologies, #72305).

Each clone was further amplified sequentially in 96-, 48-, 24-, and six-well plates and T25, T75 flask before being analyzed individually to assess the genomic edition by Sanger sequencing after DNA extraction.

#### DNA isolation of clones

DNA isolation was performed using Maxwell^®^ RSC Cultured Cells DNA Kit (Promega, #AS1620) according to the manufacturer’s recommendations using Maxwell^®^ RSC Instrument (Promega, #AS4500). DNA was recovered with 100 µL of elution buffer and measured with QuantiFluor^®^ ONE dsDNA System (Promega, #E4871). DNA was stored at -80°C until use.

#### GFP expression in control and K.O. cell lines

To help with the growth analysis, co-culture, video-microscopy, and viability measurement of cancer cells (both control and MUC2 K.O. cell lines) were further modified to express GFP under CMV promoter. The modification of cells was done using Cyagen EGFP lentivirus (Cyagen, #LV-EGFP-0102, lot#140224LVT02) as per the manufacturer’s recommendations. After culture, cells expressing GFP were further purified by cell sorting in bulk, as previously described, where GFP fluorescence was acquired with 488-nm blue laser and 530/30-nm emission filter. During cell sorting, a four-way sort mask was applied. To ensure maximum purity, the cells were serially sorted three times prior to analysis and use.

### Capillary western blot

Capillary western blot (CWB) was performed for the analysis of E-cadherin, N-cadherin, and vimentin to see the potential EMT modification of the MUC2 K.O. cells. After culture, 5 × 10^6^ cells were washed with DPBS and lysed with 400 µL of RIPA Lysis and Extraction Buffer (Thermo Fisher Scientific, #89900) complemented with Halt™ Protease Inhibitor Cocktail (Thermo Fisher Scientific, #78430) and sonicated for 30 s. Cell debris was removed by 30 min of centrifugation at 14,000*g*. Supernatants containing protein extracts were kept at -20°C until use. Protein concentration was assessed using Pierce BCA protein assay kit (Thermo Fisher Scientific, #23225). Capillary western blot was done using a Wes system (protein simple) with 12–230 kDa Separation Module, 8 × 25 capillary cartridges (Protein Simple, #SW-W004), EZ Standard Pack 2 (Protein Simple, #PS-ST02EZ-8), and anti-mouse detection module (Protein Simple DM-002). Mouse anti-human E-cadherin (R&D systems, #MAB1838) diluted at 0.5 µg/mL, mouse anti-human N-cadherin (Novus Biologicals, #NBP1-48309) diluted at 1 in 10, mouse anti-human vimentin (Novus Biologicals, #NBP1-92687) diluted at 1 in 25, and anti-β-actin (Licor, #926-42212) diluted at 1 in 100 were used as primary antibodies.

Analysis was done using compass for simple western (ProteinSimple, V5.0.0), and area of histogram peaks were used for quantification. All western blot analysis were normalized for β-actin expression.

### PBMC isolation, enrichment, activation, and staining

#### PBMC isolation

Whole blood from healthy donors collected in Sidra medicine under the IRB 1500815-2 was collected in EDTA tubes (BD biosciences, #366643) and immediately processed for PBMC isolation. The blood was diluted to 1:1 with DPBS (Gibco, #10010-031), and gradient dilution was performed with SepMate™-50 (IVD) tubes (Stem Cell Technologies, #85450) containing 15 mL of Lymphoprep (Stem Cell Technologies, #07801), according to the manufacturer’s recommendations. Briefly, cells were centrifuged in SepMate tube for 10 min at 1,200*g*. Supernatant and PBMCs were collected in a fresh tube. PBMCs were then rinsed twice with DPBS by centrifugation for 8 min at 300*g*. PBMCs were then used for magnetic bead enrichment of T and NK cells as described below.

#### PBMC enrichment

PBMCs isolated as previously described were further enriched in T cells and NK cells by elimination of CD14 and CD19 positive cells using MACS cell separation according to the manufacturer’s recommendations. Briefly, cells in 80-µL buffer per 10^7^ cells and 20 µL of CD14 microbeads (Miltenyi, #130-050-201) and CD19 microbeads (Miltenyi, #130-050-301) were incubated for 15 min at 4°C. The cells were washed with 2 mL of buffer and centrifuged for 8 min at 300*g*. The positive fraction was removed by separation on LS columns (Miltenyi, #130-042-401) on QuadroMACS separator (Miltenyi, #130-091-051) with three washes with 3 mL of buffer. Non-labeled cells were then pelleted and stored at -80°C in 90% FBS + 10% DMSO until use.

#### T-cell activation

T cells in the enriched fraction of PBMCs were thawed and activated prior to co-culture, in 2D or 3D, with cancer cells using Dynabeads™ Human T-Activator CD3/CD28 (Gibco, #1132D) as recommended by the manufacturer. Enriched and activated PBMCs are referred to as E/A-PBMCs.

#### T-cell staining for video microscopy

When performing live imaging, enriched/activated PBMCs were stained with CellTracker™ Red CMTPX Dye (Thermo Fisher Scientific, #C34552) according to the manufacturer’s recommendations prior to co-culture.

### 2D co-culture protocol and analysis

Co-culture in 2D was performed as sketched in **Supplementary Figure S2A**. A total of 500 GFP cancer cells (HT-29 and LS-174T control (CTRL) or MUC2 K.O.) were seeded per well in a 96‐well plate and cultured for 48 h in 200 µL of complete media. After removing 100 µL per well, various numbers ranging from 500 to 10,000 activated/enriched PBMCs were re-suspended in 100 µL of fresh complete media and then seeded on top of the GFP cancer cells. GFP was assessed by fluorescence intensity measurement by well scan from the bottom with excitation at 494 nm and emission at 517 nm on Ensight plate reader (PerkinElmer, #HH34000000).

### 3D co-culture protocol

Co-culturing in 3D was performed as sketched in **Supplementary Figure S2B**. A total of 2,000 cancer cells per well (CTRL or K.O.) were seeded in a low-adherence Nunclon Sphera 96U well plate (Thermo Fisher Scientific, #174925) and allowed to form spheroids for 120 h (5 days) in 200 µL complete media. On day 5, 100 µL of media was removed (without disturbing the spheroids), and 4,000 enriched/activated PBMCs were added in each well. GFP expression was monitored using GFP cancer cells on Ensight plate reader as previously described. Alternatively, GFP cancer cells and enriched/activated PBMCs stained with CellTracker™ Red were used for imaging over 48 h on CellDiscoverer 7 (CD7, Zeiss) equipped with an incubation chamber set at 37°C and 90% humidity. For flow cytometry analysis, IN and OUT fractions were separated and stained and analyzed as described below.

### IN and OUT fraction isolation

OUT and IN fractions were separated after 2 days of co-culture. OUT and IN compartments were isolated by first pooling the 30 coculture wells in FACS tubes (falcon, #). Spheroids were gently resuspended and left to sediment to the bottom of the tube. Supernatant cell suspension constituted the non-infiltrating immune cells (=OUT). These steps were repeated two times with PBS to wash the remaining spheroids (=IN) from the non-infiltrating immune cells. IN fraction was imaged with CD7 at this stage. Alternatively, both IN and OUT fractions were then trypsinized for 30 min to obtain a single-cell suspension, and both fractions were strained on a 40-um strainer (Falcon, #08-771-23) and further analyzed by flow cytometry.

### Flow cytometry analysis

Previously isolated single-cell suspensions from IN and OUT fraction were stained for flow cytometry analysis. LIVE/DEAD^®^ Fixable Aqua stain (Thermo Fisher Scientific, #L34957) was used as per the manufacturer’s recommendation. Then, unspecific sites were saturated by incubation for 30 min with blocking media consisting of DPBS completed with 5% FBS and 2% bovine serum albumin. After removing the blocking media, spheroids were incubated for 45 min with primary antibodies indicated in **Table 4** and diluted in blocking media.

**Table 4:** Antibodies used for flow cytometry and flow cytometer analysis setup.

Antibody	Clone	Manufacturer	Catalogue number	Laser wavelength	Band pass of analysis
Epcam-AF488	323/A3	Invitrogen	MA5-38713	488	530/30
CD45-PE-Texas Red	HI30	Invitrogen	MHCD4517	488	615/24
CD56-PE-Cy5	1B7	Invitrogen	15-0567-42	488	675/30
CD4-SB600	SK3	Invitrogen	63-0047-42	405	615/24
CD8a-SB436	SK1	Invitrogen	62-0087-42	405	445/45
Live/dead (fixable aqua D)		Invitrogen	L34966	405	530/30

After staining, the cells were rinsed two times with staining media and resuspended in a final volume of 100 µL. Furthermore, 80 µL was acquired on Acea Novocyte (Acea, #2010050) on low speed and analyzed with Novoexpress software (Acea). Compensations were calculated using UltraComp eBeads™ Compensation Beads (Thermo Fisher Scientific, #01-2222-42). Fluorescence minus one (FMO) was used to define the positive/negative cutoff for the gating strategy presented in **Supplementary Figure S3**.

### OCT embedding and cryosection

A total of eight to 12 spheroids of specified age and culture method were pulled together in one well of a spherical 96-U well plate. Media was removed completely, trying not to disturb the spheroids. OCT was then poured over the spheroids and allowed to solidify at -20°C. Sections of 10 µm were cut using a cryostat (Leica biosystem, #CM3050S), and the sections were attached onto Superfrost™ plus gold slides (Epredia, #FT4981IGLPLUS-001) and stored at -20°C until staining.

### Clonogenic assay

Clonogenic assay was performed as previously published (17). Briefly, cells were diluted at 100 cells per milliliter for HT-29 and 200 cells per milliliter for LS-174T and seeded 50 and 200 cells, respectively, for HT-29 and LS-174T CTRL of MUC2 K.O. in six-well plates. Whole wells were first acquired on CellDiscoverer 7 (Zeiss) at a magnification of 5× before colony counting.

### Immuno-fluorescence staining and confocal imaging

The slides were allowed to warm up at room temperature for 15 min prior to staining. The area containing spheroids was surrounded with PAP pen (Sigma, #Z377821), and unspecific sites were saturated by incubation for 30 min with blocking media consisting of DPBS completed with 5% FBS and 2% bovin serum albumin. After removing the blocking media, the spheroids were incubated for 45 min with primary antibody diluted in blocking media (mouse anti-human MUC2 antibody (CCP58) (Novus #NBP2-25221) diluted at 1:100 or rabbit anti-human Muc5B (Thermo Fisher Scientific, #PA5-82342) diluted at 1:25 or mouse anti-human Muc5AC (Thermo Fisher Scientific, #MA5-12178)) (**Table 5**). After the removal of primary antibody and two rinses of the spheroids with blocking media, 30 min with secondary antibody diluted at 1:200 in blocking media was performed (Alexa Fluor 488 (AF-488) goat anti mouse IgG (H&L) antibody (Thermo Fisher Scientific, #A-11001) or AF-488 goat anti-mouse IgG1 (Thermo Fisher Scientific, #A-21121) or AF-488 Goat anti Mouse IgG (H+L) (Thermo Fisher Scientific, #A-11008). Finally, after two rinses with blocking media, the cover slides were mounted with SlowFade™ Gold Antifade Mountant with DAPI (Thermo Fisher Scientific, #S36942).

**Table 5:** Antibodies used for immunofluorescence.

Antibody	Manufacturer	Catalogue number	Dilution
Mouse anti-hMUC2	Novus	NBP2-25221	1:100
Mouse anti-hMuc5AC	Thermofisher Scientific	MA5-12178	1:25
Mouse anti-hMuc5B	Thermofisher Scientific	37-7400	1:200
Mouse anti-hCD45-PE	BD Biosciences		1:50

Immunofluorescent-stained spheroids were imaged on Zeiss confocal microscope LSM780 (Zeiss) using 405-nm laser for DAPI analysis and in-tune laser at 490 nm for AF-488. Z-stacks of five were acquired, and the maximum intensity projection is represented.

### CD7 imaging

To study the effect of E/A PBMC’s on HT-29 and LS-174T CTRL GFP and MUC2 K.O., GFP cells were imaged for 48 h using Zeiss Cell discoverer 7 (CD7) imaging system, where E/A PBMCs were stained with cell tracker red CMTPX dye (Thermo Fisher Scientific, #C34552) following the manufacturer’s instructions. Axiocam 506 with objective lens magnification of ×5 was used, and the detection wavelengths were 470 nm for EGFP, 570 nm for AsRe2, and oblique for brightfield. Image acquisition to quantify the roundness and growth of both HT-29 and LS-174T CTRL GFP and MUC2 K.O. GFP cells were also performed using CD7 with the same optical settings and laser detectors. The roundness of both HT-29 and LS-174T CTRL GFP and MUC2 K.O. GFP cells was quantified using Zen Blue software to measure the area (µm^2^) and the perimeter (µm). The formula used to calculate the roundness of a spheroid is roundness = $\frac{{\text{perimeter}}^{2}}{4\pi }\;\times \text{ area}$ .

### Distance and velocity measurement using TrackAnalyzer tools

To evaluate the distance covered per minute and cell velocity, cells were seeded at a density of 200,000 cells per well in two-well chamber slides (Labtech, #154461). Following an 8-h adhesion period, cell movement was recorded for 2,500 min (41 h), with images captured every 20 min using a ×5 magnification lens on CellDiscoverer 7 (Zeiss). Velocity and distance covered by individual cells were analyzed using the Mosaic Toolbox in Fiji (10). A total of 20 cells per cell type were analyzed across two distinct fields.

### Imaris analysis

Spheroid growth and post-rinse analysis of HT-29 and LS-174T CTRL GFP and MUC2 K.O. GFP was analyzed by measuring its area (µm^2^) using Imaris V 9.0.1 software. EGFP channel was used as a source to analyze the spheroids for both cell lines, and the same parameters were used for the CTRL and MUC2 K.O.

### Plate reader analysis

GFP expression was analyzed on plates for both 2D and 3D culture setup using EnSight Multimode Plate Reader (PerkinElmer, #HH34000000). GFP was acquired by bottom excitation at 485-nm and emission analysis at 519-nm wavelength. A well scan was performed of 10 × 10 in round-shaped area with a distance of 0.75 between measurement for 2D and 3 × 3 in round-shaped area with a distance of 0.2 between measurements for 3D (centered around the U-bottomed well). Average intensity was measured for each well.

### Whole-genome sequencing and analysis

Library construction and sequencing was performed at the Sidra Clinical Genomics Laboratory Sequencing Facility. DNA was quantified using the Quant-iT dsDNA Assay (Invitrogen) on the FlexStation 3 (Molecular Devices). The library was constructed from 250 ng of DNA with the Illumina TruSeq DNA Nano kit. Library quality and concentration were assessed using the DNA 1k assay on a PerkinElmer GX2 and qPCR using the KAPA Library quantification kit on a Roche LightCycler 480 II. Genomic libraries were sequenced with paired-end 150-bp Novaseq 6000 systems (Illumina) following the manufacturer’s recommended protocol to achieve a minimum average coverage of 30× for each sample. Quality-passed reads were aligned to the human reference genome GRCh38 using BWA. Raw data are available at the European nucleotide archive (ENA) under accession number PRJEB82525 (ENA Browser).

WGS sequencing was performed using Illumina NovSeq 6000 platform for 30× target. Reads were mapped to the human reference genome hg38 using BWA (v.0.7.12) (20). Adaptor trimming was performed using the tool trimadap (v.0.1.3) (21). Variants calling was performed by calling the mutation from the modified samples against the control. SNP variants calling was performed using mutect2 from GATK (4.1.0.0) (22) and somatic small insertions and deletions (indels) using strelka2 (2.9.10) (23). FilterMutectCalls from GATK and FiNGS filter (v1.7.1) were used to filter false-positive variants. MAF file was generated using vcf2maf (v1.6.18) (24).

The comparison results between HT-29 CTRL and HT-29 MUC2 K.O. are available in **Supplementary Table S1**, and the comparison results between LS-174T CTRL and LS-174T MUC2 K.O. are available in **Supplementary Table S2**.

### RNA seq analysis

#### mRNA sequencing

Library construction and sequencing was performed at the Sidra Clinical Genomics Laboratory Sequencing Facility. The sample integrity and concentration of the total RNA were controlled and measured using the standard sensitivity RNA assay on the Perkin Elmer Caliper Labchip GXII. Furthermore, 500 ng of total RNA was used for library preparation using the Illumina Truseq Stranded mRNA kits. To obtain mRNA libraries, poly-A RNA selection was performed using the Oligo-dT magnetic bead system, followed by fragmentation, first-strand synthesis using Superscript IV, and second strand synthesis. The cDNA obtained after reverse transcription is then ligated with IDT for Illumina UD Indexes and amplified for 10 cycles. Library quality and concentration were then assessed using the DNA 1k assay on Perkin Elmer GX2. Library quality and concentration were then assessed using the LabChip High Sensitivity assay on a Perkin Elmer GX2 and by qPCR using the KAPA Library quantification kit on a Roche LightCycler 480 II. The RNA libraries were sequenced with paired-end 150 bp on Novaseq 6000 system (Illumina, USA) following the manufacturer’s recommended protocol at a depth of 40 million reads per sample.

Single samples were sequenced across multiple lanes, and the resulting FASTQ files were merged by sample. All the samples that passed FastQC (v. 0.11.8) were aligned to the reference genome GRChg38 using STAR (v. 2.6.1d) (18). BAM files were converted to a raw counts expression matrix using HTSeq-count (v. 0.9.1) (19).

#### Data processing and normalization

Quality control (QC) check was performed using FastQC module (Python v.2.7.1, FastQC v.0.11.2) in the raw data. Adaptor sequencing trimming was run using Flexbar (v.3.0.3) using Illumina primers FASTA file. Then, alignment of the reads to human reference genome GRCh38.93 was performed via Hisat2 (v.2.1.0) using SAMtools (v.1.3). QC was performed to confirm alignment quality and paired-end mapping overlap (Bowtie2, v.2.3.4.2). Finally, read count for each gene was created using featureCounts function of subreads (v.1.5.1). Gene count was normalized using R package EDASeq (Exploratory Data Analysis and Normalization for RNA-seq) (v. 2.34.0) to correct for within-lanes effect (GC content) and between lanes effect (sequencing depth). Then, quantile normalization was performed on the resulting values using R package preprocessCore (v.1.62.1) and then log2-transformed. All downstream analyses were done using R programming software (v. 4.3.1, or later). Principal component analysis (PCA) was performed to evaluate global changes between samples based on gene expression using “prcomp” function, and data was plotted using R CRAN package ggplot2 (v. 3.4.2). For data visualization, ggplot2 (v. 3.4.2) and ComplexHeatmap (v.2.16.0) were used for boxplot and heatmap plots, respectively.

#### Differentially expressed genes

Differentially expressed gene (DEG) analysis between groups was performed using log2 normalized expression matrix using R Bioconductor package limma (v. 3.56.2) (Ritchie et al., 2015) with Benjamini–Hochberg (B-H) FDR correction. Within each comparison, genes with row sum equal to zero were excluded. Overlapping DEGs between groups were visualized using R CRAN package Venn (v. 1.11) or volcano plot using ggplot2 (v. 3.4.2).

#### Pathway enrichment analysis

A list of DEGs (FDR < 0.01 and logFC ≥ 1) was uploaded to Ingenuity Pathways Analysis (IPA) to get the list of enriched pathways. Raw data was downloaded from IPA into R and plotted using ggplot2 (v. 3.4.2). Only the top 20 pathways based on *p*-value were plotted.

#### Single-sample gene set enrichment analysis

Single-sample gene set enrichment analysis (ssGSEA) was performed using normalized, log2-transformed expression data to calculate enrichment score (ES) using gsva() function from R Bioconductor package GSVA (v. 1.48.2) (Hänzelmann, Castelo, and Guinney 2013). The gene set to reflect enrichment of adherent junction was downloaded from Molecular Signatures Database (MSigDB). The gene set for epithelial mesenchymal transition was obtained from Liberzon et al. (2011).

### Statistical analysis

For statistical analysis and graphical presentation, GraphPad Prism V10.1.0 (Domatics) software was used. Numerical results are given as means ± SD (*N* = sample size). The statistical significance for western blot, CWB, and Q-PCR was assessed with GraphPad with unpaired Student’s *t*-test. For CD45+ cells present in the IN or OUT fraction, paired Student’s *t*-test was used for each cell line. One-way ANOVA was also performed to compare multiple conditions against each other. The statistical significance for the comparison of gene expression and enrichment score was calculated using unpaired *t*-test using R programming function “stat_compare_means” from ggpubr package. Statistical significance was accepted for *p < 0.05, **p < 0.01, *** *p*< 0.001, and ****p < 0.0001.

## Results

### MUC2 differential expression between mucinous and non-mucinous hypermutated CRC is associated with low ICR score

Differential gene expression analysis in mucinous adenocarcinoma vs. non-mucinous adenocarcinoma showed that three mucin genes were overexpressed in mucinous cancers. MUC2 was the most significantly overexpressed (*p* = 5.38e-16, logFC = 3.50), followed by MUC5B (*p* = 5.40e-11, logFC = 2.50) and then MUC6 (*p* = 2.80e-9, logFC = 2.63) as shown in **Supplementary Figure S4**. In our analysis of the AC-ICAM cohort, we also discerned a correlation between tumor histology (mucinous *versus* non-mucinous adenocarcinoma) and their immune infiltration and activation measured by the tumors’ ICR score (1). We had therefore an inverse correlation between the ICR score and MUC2 expression, leading us to perform a functional analysis of the role of mucin 2 in the immune infiltration of CRC in an *in vitro* setting.

### MUC2 expression in CRC cell lines

**Figure 1:**
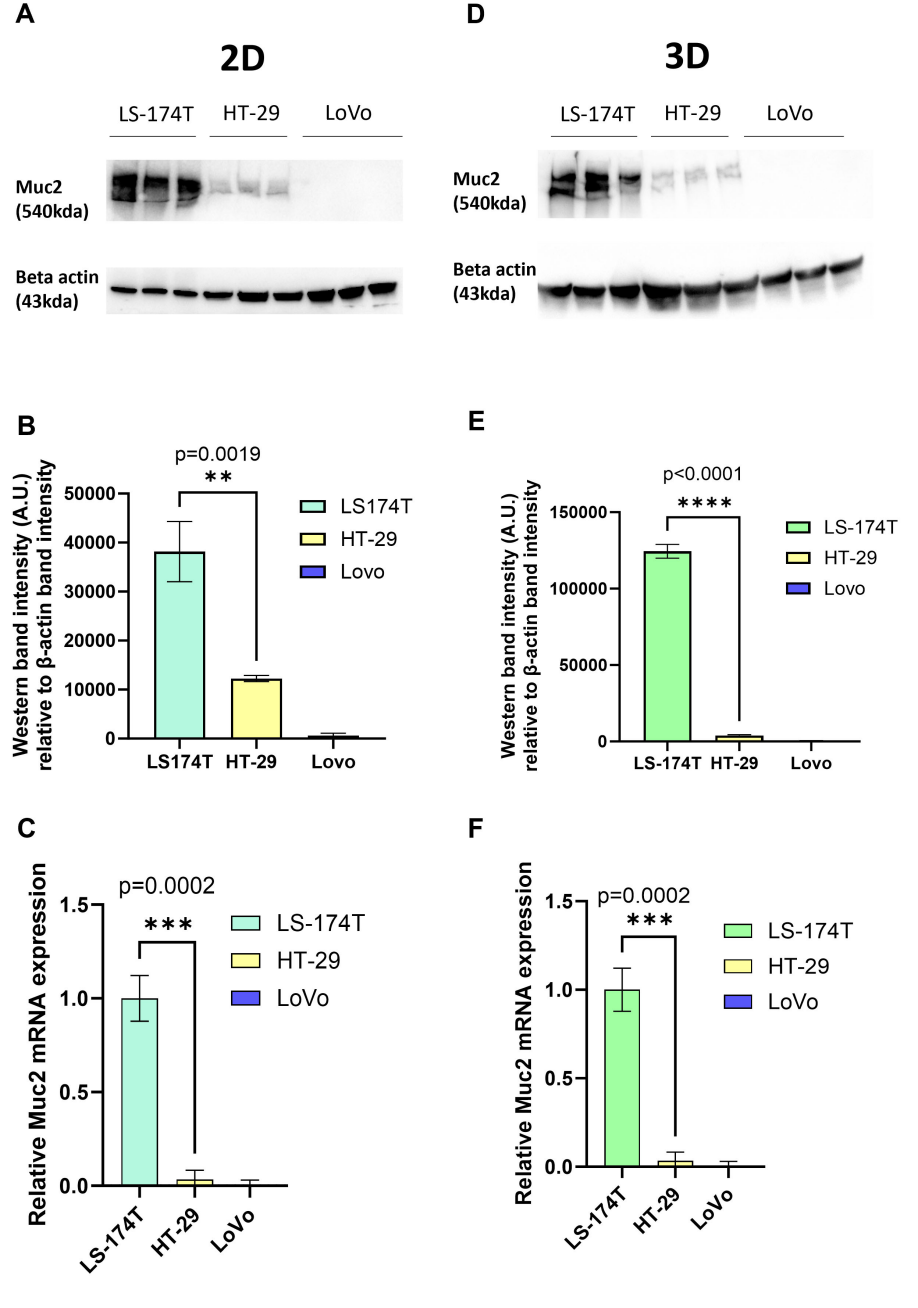
**(A)** Western blot of full-length MUC2 protein (540 kDa) and β-actin (housekeeping gene, 43 kDa) in three different cell lines LS-174T, HT-29, and LoVo grown in 2D (*N* = 3 biological replicates). **(B)** Quantification of band intensity of MUC2 protein in those three cells lines grown in 2D. MUC2 is present in abundance in LS-174T, with significantly less expression in HT-29 (*p* = 0.0019) (unpaired *t*-test), and no expression in LoVo cell line is observed. **(C)** Relative mRNA expression of MUC2 analyzed by Q-RT-PCR (*N* = 3 biological replicates) in the three cell lines grown in 2D. MUC2 expression is strongest in LS-174T compared to HT-29 (*p* = 0.0002) (unpaired *t*-test) and no expression in LoVo is observed. **(D)** Western blot of full length MUC2 protein (540kDa) and β-actin (43 kDa) in three different cell lines LS-174T, HT-29, and LoVo grown in 3D (*N* = 3 biological replicates). **(E)** Quantification of band intensity of MUC2 protein in those three cell lines grown in 2D. MUC2 is present in abundance in LS-174T, with a significantly less expression in HT-29 (*p* < 0.0001) (unpaired *t*-test), and no expression in LoVo cell line is observed. **(F)** Relative mRNA expression of MUC2 analyzed by Q-RT-PCR (*N* = 3 biological replicates) in the three cell lines grown in 2D. MUC2 expression is strongest in LS-174T compared to HT-29 (*p* = 0.0002) (unpaired *t*-test), and no expression in LoVo is observed. *p < 0.05, **p < 0.01, *** p < 0.001, and ****p < 0.0001.

In this context, we analyzed MUC2 expression in three commonly used CRC cell lines: LS-174T, HT-29, and Lovo by western blot and qPCR. As previously published (25), in 2D culture LS-174T had a significantly higher MUC2 expression compared to HT-29, while the expression of MUC2 was undetectable in LoVo (**Figures 1A–C**, **Supplementary Figures S5A, B**). A similar pattern of MUC2 expression was observed in the 3D culture of the same cell lines (**Figures 1D–F**, **Supplementary Figures S5C, D**). We therefore proceeded with the creation of MUC2 knockout mutant in the two cell lines with MUC2 expression: HT-29 and LS-174T.

### MUC2 knockout in HT-29 and LS-174T cell lines and effect on cellular behavior

Five gRNAs were designed for the knockout (K.O.) of MUC2 targeting exon 2. Each gRNA was tested on both cell lines by transfecting Cas9 ribonucleoprotein (RNP) complex by electroporation. TIDE analysis was performed for each gRNA on both cell lines. gRNA 2 was selected for HT-29 for further single-cell cloning with an approximate efficiency of 55% (**Supplementary Figure S6A**). After single-cell cloning and Sanger sequencing of each clone and analysis with ICE, HT-29 gRNA2 clone 22 was selected for further work. For this clone, knockout was achieved by the introduction of two bases after the PAM sequence on both alleles, inducing a frame shift and truncated protein production (**Supplementary Figure S6B**). This mutation was further validated by whole-genome sequencing (WGS) (**Supplementary Tables S1**, **S2**).

After TIDE analysis, gRNA 3 was selected for LS-174T for single-cell cloning with an approximate efficiency of 57% (**Supplementary Figure S6C**). After single-cell cloning and Sanger sequencing of each clone and analysis with ICE, LS-174T gRNA 3 clone 2 was selected for further work. For this clone, knockout was performed by the introduction of one base after the PAM sequence on both alleles (**Supplementary Figure S6D**). This mutation was also further validated by whole-genome DNA sequencing (**Supplementary Tables S1**, **S2**).

For both cell lines, to verify the specificity of the CRISPR edition and the absence of off-target edition, whole-genome sequencing (WGS) was performed, and no potential off-target mutations were detected in the two clones selected (**Supplementary Tables S1**, **S2**).

**Figure 2:**
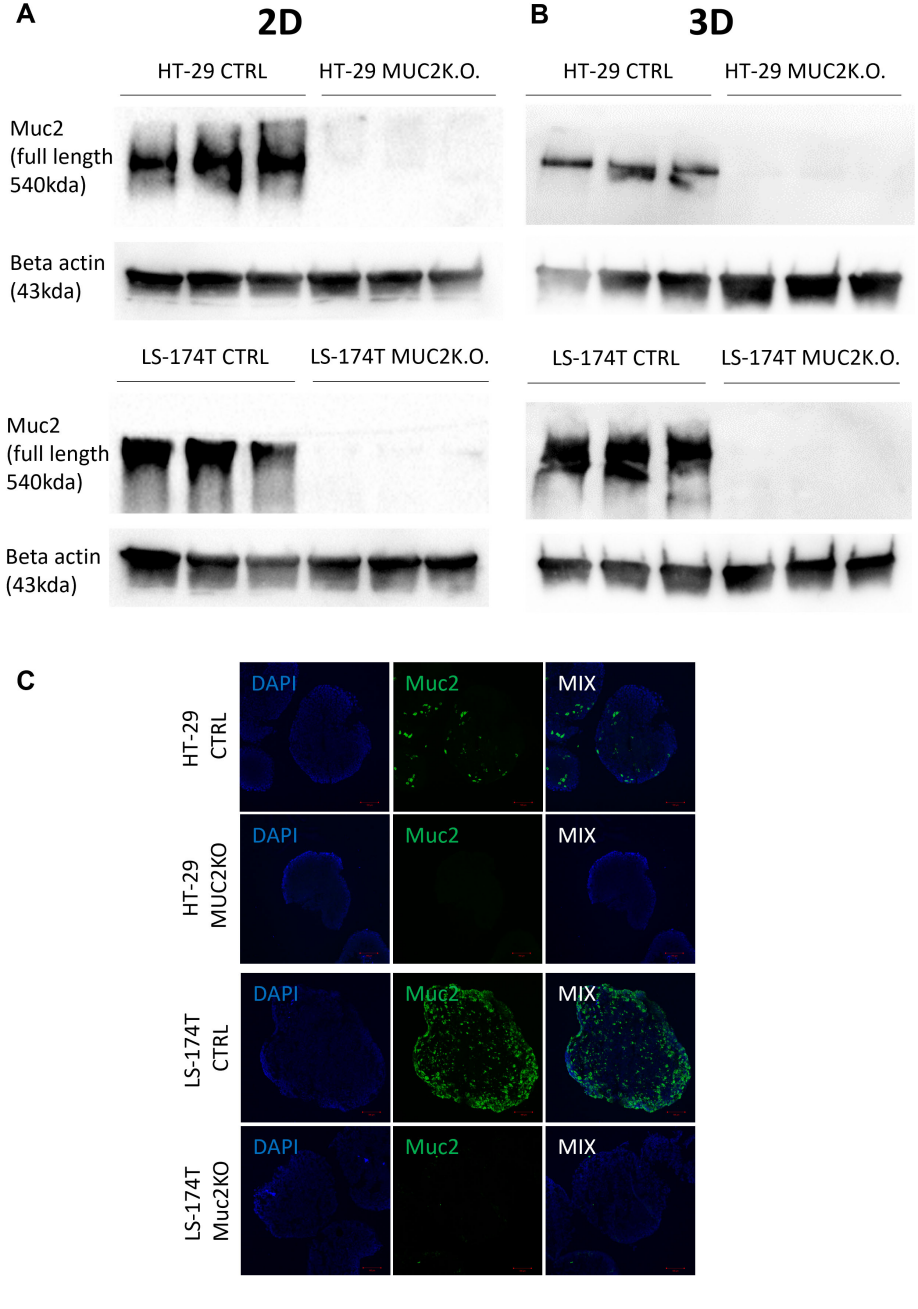
**(A)** Western blot of full-length MUC2 protein (540 kDa) and β-actin (43 kDa) in different cell lines LS-174T CTRL, LS-174T MUC2 K.O., HT-29 CTRL, and HT-29 MUC2 K.O. grown in 2D (*N* = 3 biological replicates). **(B)** Western blot of full-length MUC2 protein (540 kDa) and β-actin (43 kDa) in different cell lines LS-174T CTRL, LS-174T MUC2 K.O., HT-29 CTRL, and HT-29 MUC2 K.O. grown in 2D (*N* = 3 biological replicates). **(C)** Representative images of spheroids of HT-29 and LS-174T CTRL and MUC2 K.O. immunostaining with mouse anti-hMUC2 antibody (green) and counterstained with DAPI. Images acquired on confocal LS780. Scale bar, 100 µm.

The knockout of MUC2 expression was further validated by western blot both in 2D and 3D cultures for both cell lines (**Figures 2A, B**, **Supplementary Figures S5E–L**). Additionally, the absence of MUC2 protein was validated by immunostaining in 3D cultures for both cell lines (**Figure 2C**).

All these data, including Sanger sequencing, WGS, western blot, and immunostaining, together demonstrated the knockout of the MUC2 protein in both HT-29 and LS-174T cell lines designated as HT-29 MUC2 K.O. and LS-174T MUC2 K.O. To facilitate the analysis and imaging of those clones as well as their control counterpart (CTRL), the counterpart was further modified to express GFP as described in the “Materials and methods” section.

**Figure 3:**
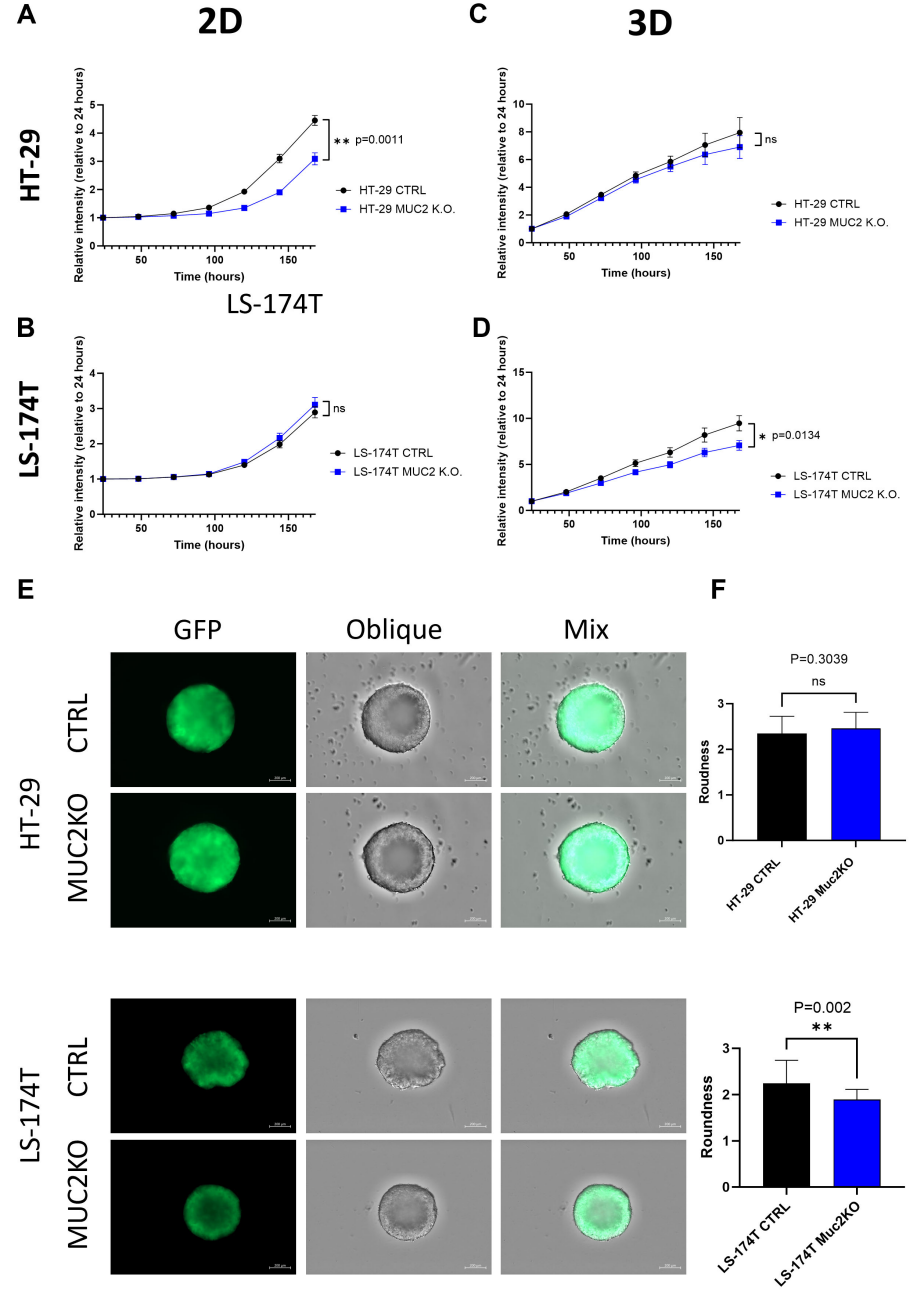
**(A)** HT-29 CTRL and HT-29 MUC2 K.O. growth in 2D culture setup monitored by plate reader for 7 days by GFP intensity (*N* = 3 biological replicates in 30 technical replicates each). HT-29 CTRL grew significantly faster than HT-29 MUC2 K.O. (*p* = 0.0011) (unpaired *t*-test). **(B)** LS-174T CTRL and LS-174T MUC2 K.O. growth in 2D culture setup monitored by plate reader for 7 days by GFP intensity (*N* = 3 biological replicates in 30 technical replicates each). No significant difference was observed (unpaired *t*-test). **(C)** HT-29 CTRL and HT-29 MUC2 K.O. growth in 2D culture setup monitored by plate reader for 7 days by GFP intensity (*N* = 3 biological replicates in 30 technical replicates each). No significant difference was observed (unpaired *t*-test). **(D)** LS-174T CTRL and LS-174T MUC2 K.O. growth in 2D culture setup monitored by plate reader for 7 days by GFP intensity (*N* = 3 biological replicates in 30 technical replicates each). LS-174T CTRL grew significantly faster than LS-174T MUC2 K.O. (*p* = 0.0134) (unpaired *t*-test). **(E)** Representative images of day 5 spheroids acquired with a Zeiss CD7 fluorescent microscope of GFP and oblique of spheroids of HT-29 and LS-174T CTRL or MUC2 K.O. Scale of 200 µm. **(F)** Roundness analysis of HT-29 (*N* = 13 each, biological replicates) and LS-174T (*N* = 25 each, biological replicates) of CTRL and MUC2 K.O. performed by analysis with Zen blue software of area and perimeter of each spheroid. No significant difference is observed between HT-29 CTRL and HT-29 MUC2 K.O., but LS-174T MUC2 K.O. is significantly rounder than LS-174T CTRL (unpaired *t*-test). **p < 0.01; ns, not significant.

We then further characterized our mutant cell lines in comparison to their control counterpart by measuring GFP using the GFP modified cell lines. In 2D culture, HT-29 MUC2 K.O. demonstrated a significantly slower growth than the original control counterpart (*p* = 0.0011) (**Figure 3A**). In contrast, LS-174T MUC2 K.O. showed similar growth compared to the control counterpart (**Figure 3B**). On the other hand, when cultured in 3D, HT-29 MUC2 K.O. showed no significant difference with the control counterpart (**Figure 3C**). However, this time, LS-174T MUC2 K.O. grew significantly slower than the control counterpart (*p* = 0.0134) (**Figure 3D**).

Additionally, we measured the size of the spheroids in 3D over 5 days of culture. Similarly, to the cell growth, HT-29 CTRL and HT-29 MUC2 K.O. displayed similar areas across 5 days (**Supplementary Figure S7A**), while the LS-174T CTRL spheroids area was higher than that of LS-174T MUC2 K.O. spheroids (**Supplementary Figure S7B**).

Incidentally, LS-174T CTRL formed spheroids with secondary appendix in 3D culture, while HT-29 CTRL spheroids appeared to be rounder and smoother (**Figure 3E**). Notably, LoVo cells barely formed spheroids after 5 days of culture (data not shown). Upon knockout of MUC2, the 3D morphology was not significantly affected in HT-29 (**Figure 3F**), while the roundness was significantly increased in LS-174T MUC2 K.O. compared to CTRL (*p* = 0.002) (**Figures 3E, F**) and with the secondary appendix being far less noticeable. The single-sample gene set enrichment analysis (ssGSEA) of HT-29 CTRL against HT-29 MUC2 K.O. as well as LS-174T CTRL against LS-174T MUC2 K.O. showed that while we observed an increase in the adherence junction enrichment score for the HT-29 MUC2 K.O. (*p* = 0.041), we observed a very strong decrease of the enrichment score in LS-174T upon K.O. of MUC2 (*p* = 0.047) (**Supplementary Figure S8A**). This opposite effect observed on the adherence junction genes could explain, in part, the opposite change in morphology of the two cell lines and potentially also the changes in cell proliferation in the 2D and 3D models.

Building on previous findings that MUC2 silencing promotes CRC metastasis (25), we investigated whether we observed increased migration or upregulated EMT markers in either of our cell lines. While we observed cell proliferation, little to no migration was observed in HT-29 cells with or without MUC2 even after 72 h. In both cases, no increase in migration was observed in MUC2 K.O. compared to CTRL when tested by scratch test (**Supplementary Figures S9A–D**), and none was observed by Transwell (data not shown). We also investigated EMT markers such as E-cadherin, N-cadherin, and vimentin by western blot in our control and MUC2 K.O. (**Supplementary Figures S10A–E**) and observed no difference in their protein expression with or without the presence of MUC2. We also investigated EMT genes’ signature by RNA expression between CTRL and MUC2 K.O. In HT-29 cells, we found no increase in EMT gene expression, while in LS-174T we observed a minimal but significant decrease upon knockout of MUC2 both in 2D (*p* = 0.00041) and 3D (*n* = 0.046) (**Supplementary Figures S11A, B**). Additionally, cell motility was evaluated using the TrackAnalyzer tool, measuring velocity and the average distance covered per minute. The analysis showed no significant differences in motility between CTRL cells and their MUC2 knockout counterparts in either the HT-29 or LS-174T cell lines (**Supplementary Figure S12**). However, LS-174T cells exhibited greater motility compared to HT-29 cells, regardless of MUC2 expression.

Finally, the clonogenic assay showed that no significant difference between CTRL and MUC2 K.O. cells was seen for neither of the two cell lines HT-29 and LS-174T (**Supplementary Figure S13**).

In summary, the effects of MUC2 knockout differed between the two cell lines. HT-29 cells with a MUC2 knockout grew slower in 2D culture, indicating a possible regulatory function of MUC2 in their proliferation. On the other hand, LS-174T cells with a MUC2 knockout exhibited a similar growth in 2D but with reduced proliferation in 3D culture, implying that the impact of MUC2 on cell behavior may depend on the environment. The MUC2 knockout did not result in any increase in cellular invasion, stemcellness, motility, or epithelial-to-mesenchymal transition in either the HT-29 or LS-174T cell lines.

### Effect of MUC2 knockout on killing by allogenic enriched activated PBMCs

**Figure 4:**
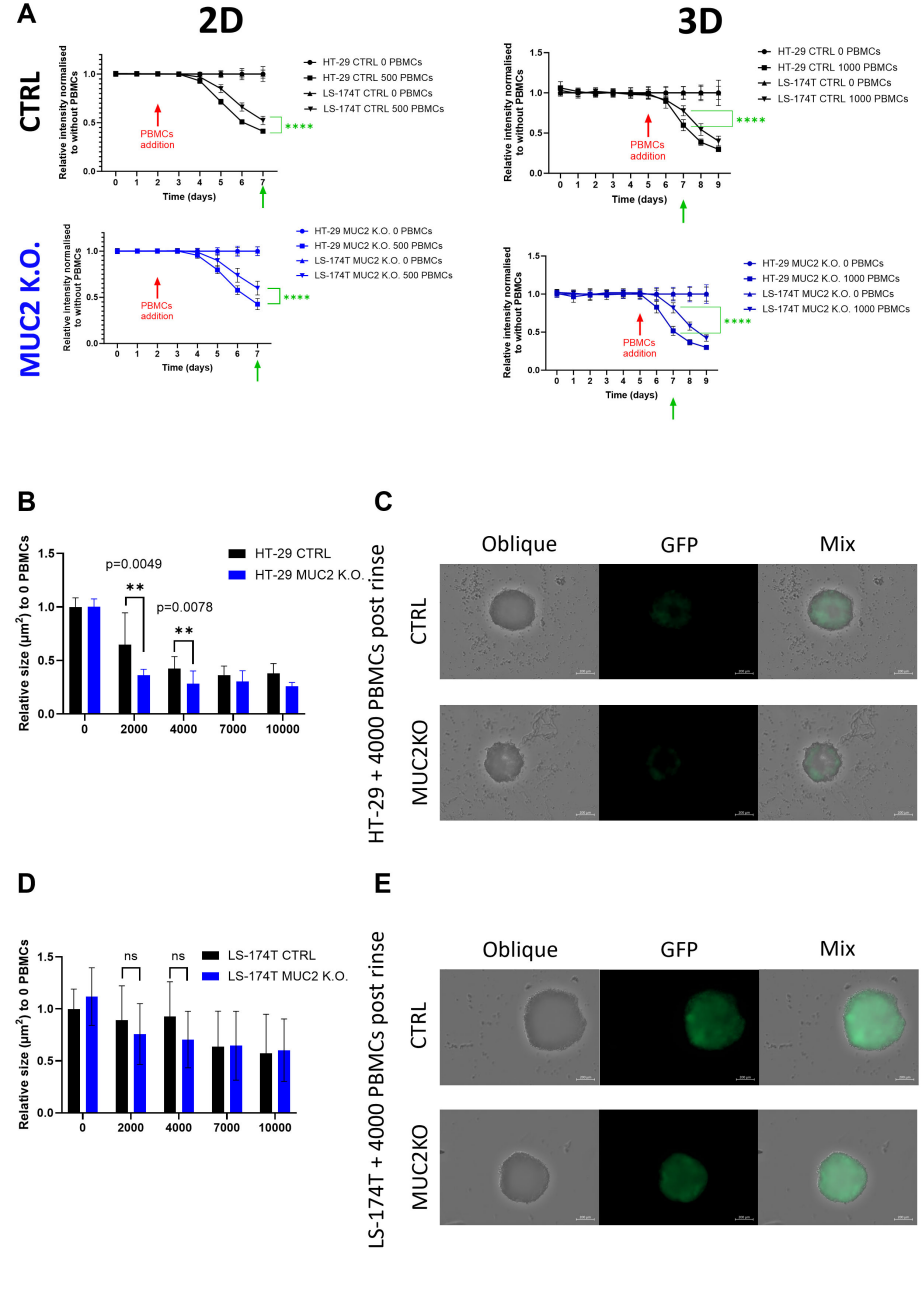
**(A)** HT-29 and LS-174T, CTRL and MUC2 K.O. growth and death monitored by GFP expression over 7 days in 2D and 9 days in 3D culture set up. 0 or 500 E/A PBMCs were added on day 2 for 2D and 0 or 1,000 E/A PBMCs were added on day 5 for 3D co-culture setup. Data are presented as relative to the same cell line without co-culture with PBMCs (0 E/A PBMCs) (*N* = 18, six technical replicates of three biological replicates). Significant differences between HT-29 and LS-174T cell lines were calculated at day 7 in 2D and 3D by Student’s *t*-test. **(B)** HT-29 CTRL and MUC2 K.O. spheroids area of IN fraction spheroids post-rinse after co-culture for 2 days with E/A PBCMs relative to the area of IN fraction after rinsing without co-culture. After co-culture with 2,000 or 4,000 PBMC, the area of IN fraction spheroids is significantly smaller in HT-29 MUC2 K.O. than in HT-29 CTRL (*N* = 12, six technical replicates of two biological replicates) (unpaired *t*-test). **(C)** Representative images of IN fraction spheroids post-rinse after co-culture with 4,000 E/A PBMCs of HT-29 CTRL and HT-29 MUC2 K.O. Images were acquired for oblique and GFP expression on Zeiss CD7, and the area was analyzed with Imaris software. Scale bar, 200 µm. **(D)** LS-174T CTRL and MUC2 K.O. spheroid area of IN fraction spheroids post-rinse after co-culture for 2 days with E/A PBCMs relative to the area of IN fraction after rinsing without co-culture. No significant difference was observed between LS-174T CTRL and MUC2 K.O. at any number of E/A PBMC co-culture (*N* = 12, two biological of six technical replicates) (unpaired *t*-test). **(E)** Representative images of IN fraction spheroids post-rinse after co-culture with 4,000 E/A PBMCs of LS-174T CTRL and LS-174T MUC2 K.O. Images were acquired for oblique and GFP expression on Zeiss CD7, and the area was analyzed with Imaris software. Scale bar, 200 µm. **p < 0.01; ns, not significant.

We first investigated if the absence of MUC2 protein would give increased susceptibility to killing by allogenic activated T cells from PBMCs enriched in T cells and NK cells (E/A PBMCs). In a 2D setup, we plated 500 GFP-expressing cancer cells and allowed them to adhere and grow for 48 h prior to the addition of varying numbers of E/A PBMCs. The number of cancer cells was monitored by GFP measurement over 5 days of co-culture. To account for the difference in growth speed documented previously between CTRL and MUC2 K.O., the results were normalized to control cells without PBMCs. Similarly, in a 3D setup, we plated 2,000 GFP-expressing cancer cells in ultralow-adherence 96-well plates and allowed them to form spheroids for 5 days prior to the addition of varying numbers of E/A PBMCs. The number of cancer cells was monitored by GFP expression measured on a plate reader for up to 4 days of co-culture. While both in 2D and 3D, we could observe a decrease in cancer cells with every increase in the amount of E/A PBMCs plated on the cancer cells, but no significant difference could be observed between CTRL and MUC2 K.O. in both cell lines (**Supplementary Figure S14A–F**), though we could identify a higher sensitivity of HT-29 cells compared to LS-174T in both CTRL and MUC2 K.O. to co-culture with 500 E/A PBMCs in 2D and co-culture with 1,000 E/A PBMCs in 3D (**Figure 4A**). Representative videos of the co-culture in 3D over 2 days are shown as supplementary data (**Supplementary Figure S15**). It shows a strong interaction of the PBMCs and a strong expansion of E/A PBMCs when co-cultured with cancer cells, yet no drastic visual differences between CTRL and MUC2 K.O. could be observed at this point. GFP measurement could not demonstrate an increase in cancer cell killing by the removal of MUC2 protein in either of the cell lines, using any of the E/A PBMC numbers, in either a 2D or 3D setting.

Overall, using plate readers, we did not discern any increase in the killing efficacy of MUC2 knockout cells by enriched activated PBMCs, in either 2D or 3D settings. We could see an effect on HT-29 MUC2 K.O only through microscopy, after rinsing the spheroids,.

### Effect of MUC2 knockout on PBMC infiltration

We further investigated E/A PBMC infiltration of the spheroids by microscopy and flow cytometry. Using the same conditions as previously discussed, we co-cultured E/A PBMCs with cancer cells. The IN and OUT fractions were then separated, and the remaining IN fraction size was evaluated by microscopy. A significant size difference was observed with HT-29, with a smaller IN fraction left with HT-29 MUC2 K.O. compared to CTRL after 2 days of co-culture with 2,000 PBMCs (*p* = 0.0049) or 4,000 PBMCs (*p* = 0.0078) (**Figures 4B, C**), while no significant difference was observed between the remaining IN fraction of LS-174T CTRL and LS-174T MUC2 K.O. (**Figures 4D, E**).

**Figure 5:**
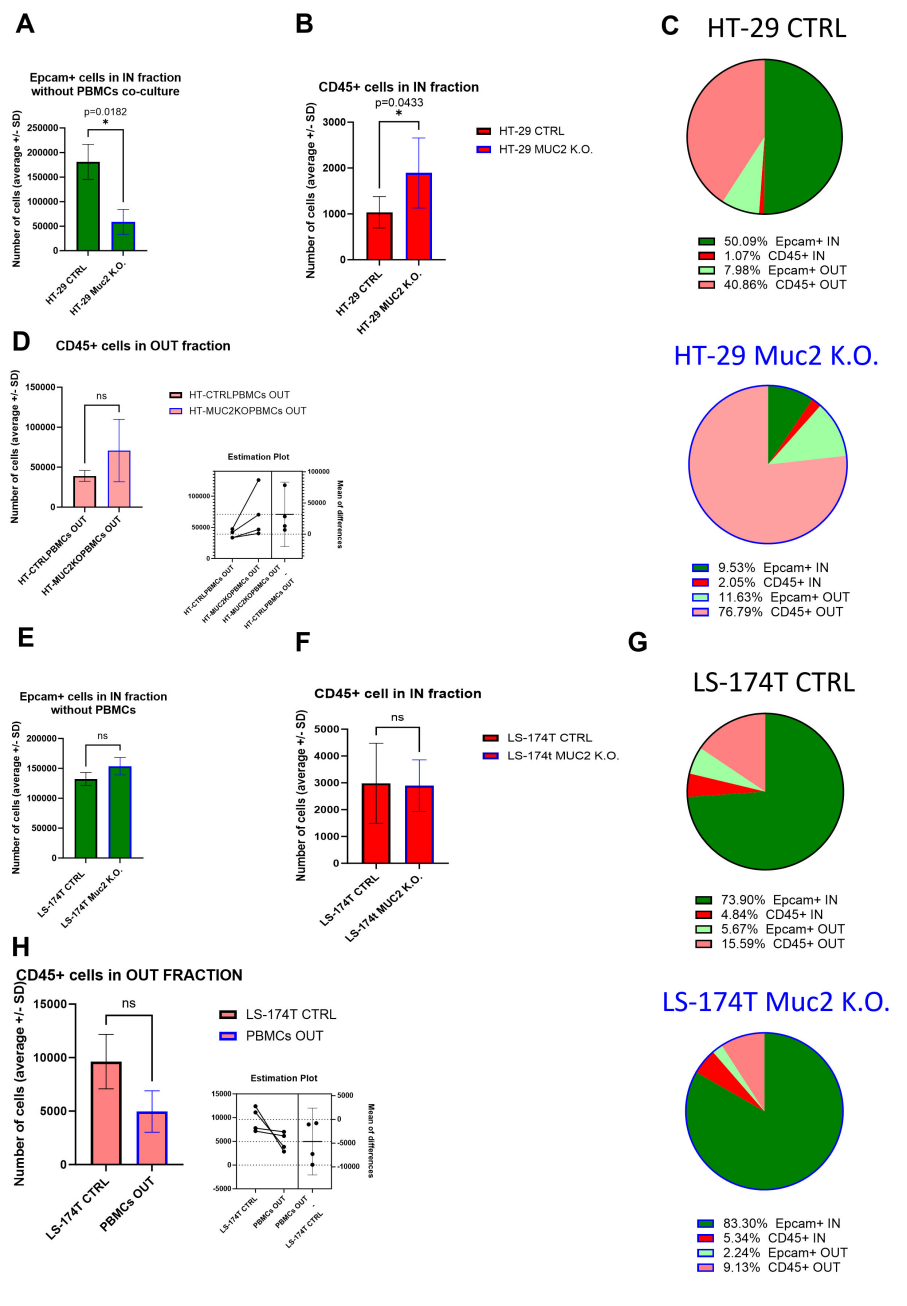
**(A)** Average number of Epcam+ cells recovered in the IN fraction after digestion, staining, and flow analysis without co-culture with E/A PBMCs. Significantly less Epcam+ cells were recovered for HT-29 CTRL than for HT-29 MUC2 K.O. (*p* = 0.0182, paired *t*-test) (*N* = 4 biological replicates). **(B)** Average number of CD45+ cells recovered in the IN fraction after digestion, staining, and flow analysis. Significantly less CD45+ cells were recovered for HT-29 CTRL than for HT-29 MUC2 K.O. (*p* = 0.0433, paired *t*-test) (*N* = 4 biological replicates). **(C)** Pie chart representation of average relative abundance of Epcam+ and CD45+ cells in the IN and OUT fraction after 2 days of co-culture for HT-29 CTRL and MUC2 K.O. (*N* = 4 biological replicates). **(D)** Average number of CD45+ cells recovered in the OUT fraction after digestion, staining, and flow analysis. No significant difference in CD45+ cells recovered after co-culture with HT-29 CTRL or HT-29 MUC2 K.O. was detected despite having systematically more CD45+ cells after co-culture with HT-29 MUC2 K.O. than with HT-29 CTRL (*p* = 0.1460, paired *t*-test) (*N* = 4 biological replicates). **(E)** Average number of Epcam+ cells recovered in the IN fraction after digestion, staining, and flow analysis without co-culture with E/A PBMCs. No statistical difference was observed in Epcam+ cells recovered between LS-174T CTRL and LS-147T MUC2 K.O. (*p* = 0.1405, paired *t*-test) (*N* = 3 biological replicates). **(F)** Average number of CD45+ cells recovered in the IN fraction after digestion, staining, and flow analysis. No difference in CD45+ cells recovered was observed between LS-174T CTRL and LS-174T MUC2 K.O. (*p* = 0.9202, paired *t*-test) (*N* = 4 biological replicates). **(G)** Pie chart representation of average relative abundance of Epcam+ and CD45+ cells in the IN and OUT fraction after 2 days of co-culture for LS174T CTRL and MUC2 K.O. (*N* = 4 biological replicates). **(H)** Average number of CD45+ cells recovered in the OUT fraction after digestion, staining, and flow analysis. No significant difference in CD45+ cells recovered after co-culture with LS-174T CTRL or LS-174T MUC2 K.O. was detected despite having systematically more CD45+ cells after co-culture with LS-174T CTRL than with LS-174T MUC2 K.O. (*p* = 0.1262, paired *t*-test) (*N* = 4 biological replicates). *p < 0.05; ns, not significant.

To further investigate the number of cells within each fraction, we performed a flow cytometry analysis of the two fractions for each cell line. When analyzing by flow cytometry the IN fraction in the absence of a co-culture with PBMCs, we recovered significantly less Epcam+ cells in HT-29 MUC2 K.O. than in HT-29 CTRL (*p* = 0.0182) (**Figure 5A**) despite a previous observation of similar growth in 3D setup between CTRL and MUC2 K.O (**Figure 3C**, **Supplementary Figure S7A**). The increase in this cell line of adherens junction expression (**Supplementary Figure S8A**) could explain, in part, this difference. Moreover, we also noticed that during filtration on a 40-µm cell strainer prior to the flow cytometry analysis, more clumps were retained with HT-29 MUC2 K.O spheroids than their CTRL counterpart (data not shown). On the other hand, with LS-174T, while the difference is not significant, we recovered slightly more Epcam+ cells in LS-174T MUC2 K.O. IN fraction than in the CTRL counterpart (**Figure 5E**). Once again, this might be due to the reduction in expression of adherens junction proteins between the cells in the MUC2 K.O. compared to the CTRL in this cell line, facilitating a more efficient dissociation. A direct comparison of the number of Epcam+ cells recovered in the IN fraction was therefore not possible. The CD45^+^ cells on the other hand should not have been affected by this adherens junction expression in the cancer cells, and we could compare the number of CD45^+^ cells recovered. We saw a significant increase of CD45^+^ cells in the IN fraction of HT-29 MUC2 K.O. compared to CTRL (**Figures 5B, C**). In contrast, no difference was observed in LS-174T (**Figures 5F, G**). Also, in the OUT fraction, while the difference is not significant, we recovered slightly more CD45^+^ cells after co-culture with HT-29 MUC2 K.O. (**Figures 5C, D**) than with the CTRL cells. Conversely, slightly fewer CD45^+^ cells were recovered with LS-174 MUC2 K.O. than with CTRL (**Figures 5G, H**). Additionally, no enrichment in any immune cell subtype was observed between CTRL and MUC2 K.O. in either cell line for the IN and OUT fractions (**Supplementary Figure S16**).

In summary, our flow cytometry analysis revealed that the knockout of MUC2 in HT-29 cells resulted in the increased proliferation of E/A PBMCs and in immune cell infiltration in the cancer cell spheroids, leading to increased cancer cell death and reduction in cancer spheroids. Conversely, such effects were not evident in LS-174T cells. This further confirms the previous observations made using microscopy (**Figures 4B–E**).

### DEG analysis of the invading PBMCs show cell cycle and interferon pathway activation in HT-29 MUC2 K.O. cells only

**Figure 6:**
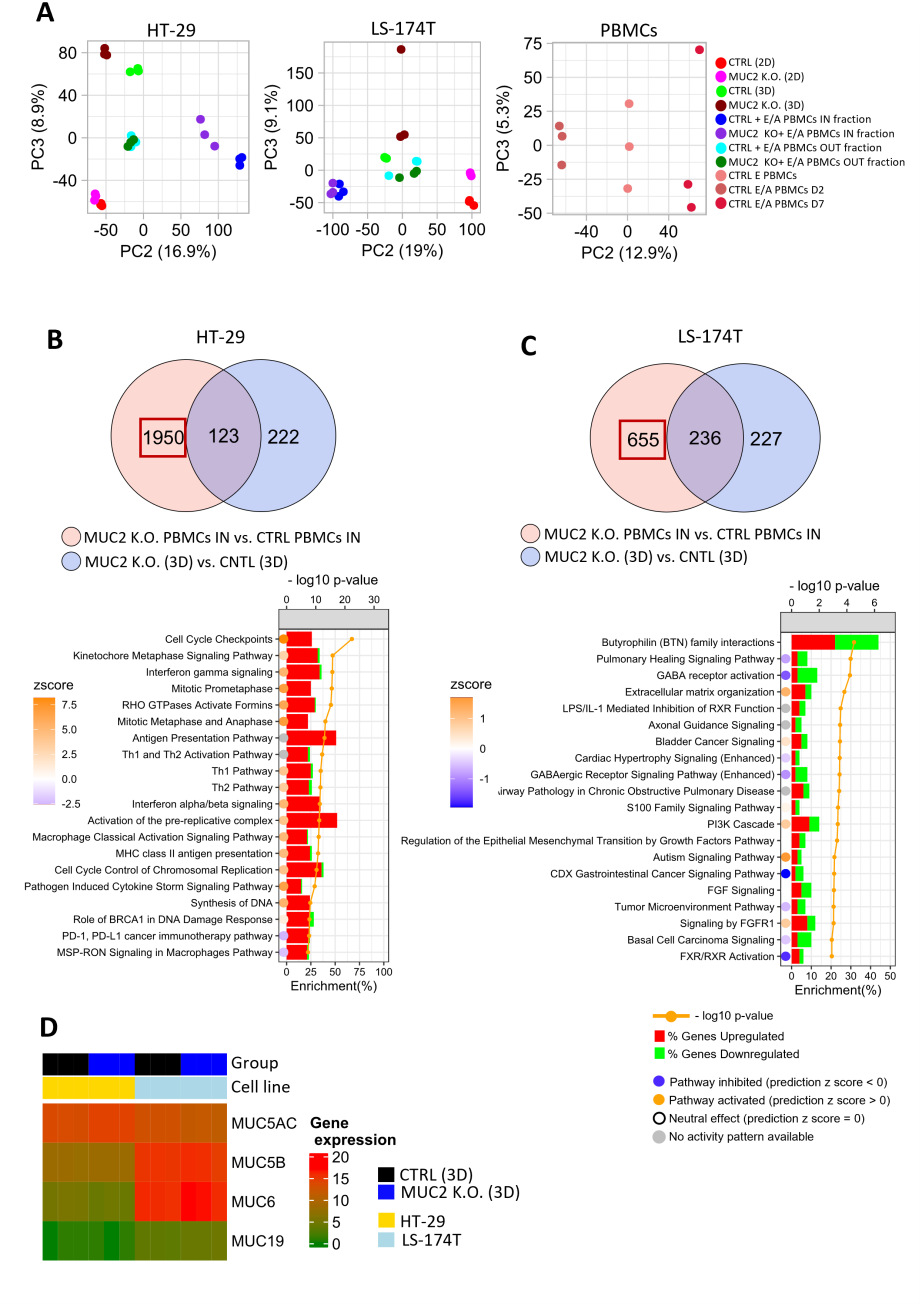
**(A)** Principal component analysis (PCA) based on gene expression profile in HT-29, LS-174T cultured in 2D and 3D, E/A PBMCs or IN and OUT fraction of co-culture of cancer cells with E/A PBMCs. **(B)** Venn diagram of DEG (FDR < 0.01, logFC >= 1) between HT-29 MUC2 K.O. + E/A PBMCs IN fraction vs. HT-29 CTRL + E/A PBMCs IN fraction (*n* = 2073 genes) and HT-29 MUC2 K.O. (3D) vs. HT-29 CTRL (3D) (*n* = 345). Pathway enrichment analysis was performed on genes reflecting DEG between PBMCs IN (MUC2) vs. PBMCs IN (CTRL) (*n* = 1,950) by subtracting the common genes. The top 20 pathways based on *p*-value were selected for plotting. A comparison of those two DEG lists allows us to see that when in contact with HT-29 MUC2 K.O., E/A PBMCs increase their cell cycle and are IFN pathway activation. **(C)** Venn diagram of DEG (FDR < 0.01, logFC ≥ 1) between LS-174T MUC2 + E/A PBMCs IN fraction vs. LS-174T CTRL + E/A PBMCs IN fraction (*n* = 891) and MUC2 K.O. (3D) vs. CNTL (3D) (*n* = 463). Pathway enrichment analysis was performed on genes reflecting DEG between PBMCs IN (MUC2) vs. PBMCs IN (CTRL) (*n* = 655) by subtracting the common genes. Top 20 pathways based on *p*-value were selected for plotting (FDR < 0.01). A comparison of those two DEG lists does not demonstrate a similar pattern with LS-174T. **(D)** The gene expression of other gel-forming mucin in HT-29 and LS-174T spheroids shows that LS-174T also have a strong expression of MUC5B, MUC6, and MUC19 compared to HT-29. **(A, B)** Histograms represent the proportion (%) of DEGs upregulated (red) or downregulated (green) in PBMCs IN (MUC2) vs. PBMCs IN (CTRL). The circles represent the pathway activation status. The blue circle indicates that the pathway is inhibited with a negative z-score, the orange circle represents that a pathway is activated with a positive z-score, the white circle represents that the pathway is neutral with zero z-score, while the gray circle indicates that the pathway activity is unknown.

To further understand the different impact MUC2 knockout has on our two cell lines, we performed RNA sequencing analysis of both cell lines with and without co-culture. The RNA seq was performed in 2D and 3D for CTRL and MUC2 K.O. cells, in addition to CTRL PBMCs (each RNA seq was performed in biological triplicates). Global transcriptional change was assessed using PCA. The results show a clear separation between CTRL-3D (green) and MUC2 K.O.-3D (brown)) in both cell lines. In HT-29 cell line, CTRL + E/A PBMCs IN fraction (blue) separates from MUC2 K.O. + E/A PBMCs IN fraction (purple), while this separation is less obvious for LS-174T. Moreover, there is a separate cluster of CTRL PBMCs (peach), CTRL E/A PBMCs D2 (control enriched/activated PBMCs at day 2) (brown), and CTRL E/A PBMCs D7 (red) (**Figure 6A**).

In conclusion, the PCA analysis revealed distinct global transcriptional changes due to MUC2 knockout in both HT-29 and LS-174T cell lines, with PCA showing a clear separation between the 3D cultures of CTRL and MUC2 K.O. cells. More importantly, HT-29 cells co-cultured with PBMCs showed a significant separation between CTRL and MUC2 K.O. conditions, unlike LS-174T, highlighting cell-line-specific responses to PBMC co-culture when MUC2 is knocked out.

We then evaluated the effect MUC2 K.O. had on each cell line. HT-29 cells grown in 2D displayed upon MUC K.O. significant upregulation of genes in pathways associated with cell cycle checkpoints. This is compatible with our observation of significantly slower cell proliferation in cells missing MUC2 expression (**Supplementary Figure S17A**). In 3D though, little difference in gene expression was observed for HT-29 (**Supplementary Figure S16B**). In LS-174T cells, while a significant number of DEGs were identified in both in 2D and 3D, no particular or significant *p*-value enriched pathways were noticed (**Supplementary Figures S17C, D**).

In order to then compare the co-cultures, we first performed the RNA sequencing of the PBMCs alone before and after activation prior to co-culture with cancer cells. We then performed RNA sequencing of the IN (part of the spheroid) and OUT (in suspension) fraction of each cell line after 2 days of co-culture. To identify genes specific to PBMCs in the cell fractions, we compared DEGs between CTRL cancer cells + PBMCs and MUC2 K.O. cancer cells + PBMCs. We then intersected this list with DEGs from comparing CTRL cancer cells alone to MUC2 K.O. cells alone in 3D culture. By removing overlapping genes, we isolated genes likely differentially expressed by PBMCs after co-culture or by cancer cells after PBMC contact (**Figure 6B**). In the HT-29 MUC2 K.O. cells followed by co-culture with PBMCs, the identified DEGs (*n* = 1,950) are involved with cell cycle checkpoints (-log10 *p*-value = 22.4, *p* = 3.98e-23), kinetochore metaphase signaling pathway (cycle progression pathway) (-log10 p-value = 15.8, *p* = 1.58e-16) and INF-g signaling (-log10 p-value=15.7, *p* = 1.99e-16) (**Figure 6B**). When the same analysis was performed with LS-174T cancer cells (*n* = 655), no significant pathway with a strong *p*-value could be detected (**Figure 6C**). Also, when analyzing the ICR score in the IN co-culture fraction, only in HT-29 did we notice a significant increase of the ICR score (*p* = 0.009) (**Supplementary Figure S18**).

We clearly identified a difference in response to co-culture with E/A PBMCs between HT-29 and LS-174T upon knockout of MUC2. While little to no effect is observed with LS-174T, a strong effect is observed with HT-29 as they become much more sensitive to killing by E/A PBMCs.

To try to understand the cause of this difference between the two cell lines, we looked at the expressions in our cell lines of mucin genes known to be gel forming: MUC2, MUC5AC, MUC5B, MUC6, and MC19. While the presence of MUC2 mRNA is seen (**Figure 6D**), due to the knockout by indel, it is not translated into functional protein in the knockout cells (no protein detected by western blot or by immunofluorescence) (**Supplementary Figure S20**). MUC5AC expression was increased in HT-29 MUC2 K.O. compared to CTRL and decreased in LS-174T MUC2 K.O. The expression of MUC5AC in both HT-29 and LS-174T (both CTRL and MUC2 K.O.) was confirmed by immunofluorescence in 3D (**Supplementary Figure S20**) and by western blot (**Supplementary Figure S21**) in 2D. While in immunofluorescence in 3D both seem to express MUC5AC at similar levels, the western blot analysis in 2D confirms the observation made in RNA seq on 2D cells, with less expression in LS-174T compared to HT-29. MUC5B was not significantly differentially expressed between CTRL and MUC2 K.O. for either cell lines, but MUC5B was much more expressed in LS-174T than HT-29 cells when analyzed in 3D by immunofluorescence (**Supplementary Figures S19**, **S20**). Indeed MUC5B was not detected in HT-29 cells (both CTRL and MUC2 K.O.) but was detected in LS-174T (**Supplementary Figure S20**). Similarly to MUC5B, MUC6 was significantly more expressed in LS-174T cells compared to HT-29 cells (*p* = 3e-04) (**Figure 6D**, **Supplementary Figure S19**). Unfortunately, no successful immunostaining could be made on our spheroids of MUC6 to validate this finding. Finally, MUC19 was also significantly more expressed in LS-174T cells compared to HT-29 cells (*p* = 0.057) (**Figure 6D**, **Supplementary Figure S19**), though the level of expression was very low compared to the other gel-forming mucins. Also, a slight increase in expression of MUC19 was observed in LS-174T MUC2 K.O. compared to LS-174T CTRL (*p* = 0.036). While both cell lines expressed MUC5AC, LS-174T expressed significantly more MUC5B, MUC6, and MUC19 than in HT-29 cells (**Figure 6D**, **Supplementary Figures S19**, **S21**).

In summary, in MUC2 knockout cells, we saw an overall higher expression of MUC5B, MUC6, and MUC19 in LS-174T compared to HT-29.

## Discussion

The research conducted in this study aimed to unravel the intricate role of MUC2, a mucin protein, in the context of colorectal cancer (CRC), with a specific focus on its influence on immune response dynamics and cellular behavior. The investigation stemmed from the observation of an inverse correlation between MUC2 expression and the immune constant of rejection (ICR) score in mucinous adenocarcinomas compared to other histological types.

In interpreting our findings from the previously published multi-omics dataset comparing mucinous and non-mucinous CRC with the ICR, two hypotheses emerge. The first suggests that mucin may serve as a barrier against bacterial invasion, akin to its role in the normal colon mucosa, subsequently deterring immune cell attraction by the intratumoral microbiome. However, our findings in the AC-ICAM study showed that there are bacterial genera associated with ICR and there are bacteria-associated prognosis; these genera were not the same.

The second hypothesis, which is not mutually exclusive with the first, posits that mucin could act as a physical barrier directly impeding immune cells from penetrating the tissues. This study focuses on this second hypothesis and seems to validate these observations *in vitro*. Specifically, the removal of MUC2 in HT-29 cells resulted in heightened immune infiltration and enhanced allogenic recognition of cancer cells.

The study employed a multi-pronged approach, utilizing MUC2 knockout (K.O.) in CRC cell lines HT-29 and LS-174T, thus shedding light on the nuanced effects of MUC2 on various cellular processes. The successful knockout of MUC2 was meticulously validated through a series of analyses, including Sanger sequencing, whole-genome DNA sequencing, western blot, and immune staining. This rigorous validation process ensured the reliability of subsequent findings and conclusions. One of the intriguing observations was the divergent impact of MUC2 knockout on the two distinct cell lines. HT-29 MUC2 K.O. cells exhibited a slower growth in 2D culture, hinting at a potential regulatory role of MUC2 in the proliferation of these cells or their interaction with the vessel. In contrast, LS-174T MUC2 K.O. cells demonstrated a comparable growth in 2D but a significantly slower growth in 3D culture, suggesting a context-dependent influence of MUC2 on cellular behavior. Further exploration into the molecular mechanisms revealed an opposite effect on tight junction genes in the two cell lines. This differential gene expression may underlie the observed variations in cell morphology and growth patterns, emphasizing the complexity of mucin involvement in CRC.

Importantly, the study delves into the impact of MUC2 on the immune response. Sensitive techniques such as quantitative microscopy and flow cytometry analyses unveiled a significant increase in immune cell infiltration in HT-29 MUC2 K.O. We also observed increased activation and proliferation of the T cells as they seem to have better access to the HT-29 cells, leading to increased allogeneic immune rejection. This strongly suggests that MUC2 acts as a physical barrier hindering immune cell recognition and activation.

The RNA sequencing data provided further insights, indicating that pathways associated with cell cycle and IFN-y signaling were upregulated in PBMCs co-cultured with HT-29 MUC2 K.O. Our data also shows an upregulation of the cell cycle checkpoints. We believe that this signature is due to the cancer cells in this remaining IN fraction as it was previously demonstrated that IFNs can indeed affect cell proliferation in tumor cells both by prolonging or blocking the cell cycle (26–29). This finding aligns with the enhanced immune infiltration observed in these conditions, reinforcing the notion that MUC2 impedes immune cell access to cancer spheroids.

The study also considered the expression of other gel-forming mucins, revealing that while MUC5AC expression increased in HT-29 MUC2 K.O., MUC5B, MUC6, and MUC19 showed no significant differences and were significantly less expressed in HT-29 than in LS-174T. Therefore, we hypothesize that the absence of increased immune infiltration observed in LS-174T MUC2 K.O. is due to the presence of those other mucin proteins in the ECM, in line with the presence of MUC5B and MUC6 being significantly over-expressed (with lower fold change and *p*-value than MUC2) genes in mucinous adenocarcinoma compared to other carcinomas. An important observation in our study is that MUC2 expression is inherently lower in HT-29 cells compared to LS-174T cells. Despite this, the absence of MUC2 has a more pronounced impact on HT-29 cells. MUC2 serves as the primary gel-forming mucin in the intestinal epithelium, whereas MUC5AC and MUC6 are predominantly expressed in the stomach, and MUC5B plays a pivotal role in the respiratory tract. Consequently, the removal of MUC2 as the major gel-forming mucin in the intestinal environment in the absence or lower expression of alternative mucins such as MUC5B or MUC6 leads to a loss of physical barrier played by MUC2. This is further supported by the least significant differences in the expression of MUC5B and MUC6 between mucinous and non-mucinous CRC in our patient cohort, underscoring their secondary role in this specific context.

In conclusion, this research contributes valuable insights into the multifaceted role of MUC2 in CRC. The divergent effects observed in HT-29 and LS-174T cells underscore the importance of considering cellular context and heterogeneity in studying mucin functions. These observations suggest that MUC2 and potentially other mucins play a role in creating a barrier that limits immune cell access to cancerous tissues. This barrier function may prevent immune cell recognition and activation within the tumor microenvironment. The results of our study highlight the complex interplay between mucins, immune response, and the intricate dynamics of the tumor microenvironment in colorectal cancer. Further exploration of these mechanisms is warranted to elucidate the specific molecular pathways involved and to inform potential therapeutic strategies to manipulate mucin-related interactions in cancer immunity.

## Data Availability

All data produced in the present study are available as supplementary data. WGS raw data are available at the European nucleotide archive (ENA) under Accession number PRJEB82525.

## Supplementary material

Supplementary Figure 1 Relative localization and orientation of the five gRNAs design for knockout of MUC2 on exon 2 and the primers used for PCR and Sanger sequencing.

Supplementary Figure 2
**(A)** Schematic of the co-culture and analysis procedure used for 2D experiments. A total of 500 cancer cells were seeded on each well of a 96-well plate at day 0 and allowed to settle and grow for 2 days. On day 0, enriched PBMCs were thawed and activated for 2 days. Various numbers of enriched/activated cells are then seeded on top of the cancer cells at day 2. GFP expression is recorded every day from day 0 to 7 on EnSight plate reader. **(B)** Schematic of the co-culture and analysis procedure used for 3D experiments. A total of 2,000 cancer cells per well are seeded on low-adherence U-bottomed well plates. Spheroids are allowed to be formed for 5 days. On day 3, enriched PBMCs were thawed and activated for 2 days. Co-culture was then performed for 2–4 days. GFP expression was monitored on a plate reader from day 0 to 9. A video-microscopy of the co-culture was performed between days 5 and 7. If microscopy analysis of remaining IN fraction is performed, spheroids were collected at day 7. For flow cytometry, IN and OUT fractions are separated; then, for flow cytometry, each fraction is treated with trypsin prior to staining and analysis.

Supplementary Figure 3 Gating strategy for flow cytometry analysis. Cells were first selected on FSC-A/SSC-A parameters. Singlets were selected on SSC-H/SSC-A and FSC-H/FSC-A parameters. Live cells were selected on the base of expression of SSC-A/V2-A (Ex 405-Em530/30). Epcam+ and CD45+ cells were then selected from living cells based on B1-A/B2-A (Ex 488-Em615/24). CD45+ cells were further distinguished for CD4+/CD8a+ expression based on V1-A (Ex405-Em445/45)/V4-A (Ex405-Em615/24), and CD56+ cells were analyzed based on SSC-A/B4-A (Ex488-Em615/24).

Supplementary Figure 4 Volcano plot of the DEG using limma between mucinous *versus* all other histological classifications in hypermutated samples of the AC-ICAM cohort. The most significant gene expressed in mucinous samples with the higher fold change is MUC2 (*p* = 5.38e-16, logFC = 3.50). Two other mucins are identified as upregulated in mucinous samples: MUC5B (*p* = 5.40e-11, logFC = 2.50) and MUC6 (*p* = 2.80e-9, logFC = 2.63). Red dots represent genes with *p*-value <0.005 and gray dots for genes with *p*-value >0.005.

Supplementary Figure 5
**(A, B)** Full uncropped western blot of full-length MUC2 protein (540 kDa) **(A)** and β-actin (housekeeping gene, 43 kDa) **(B)** in three different cell lines LS-174T, HT-29, and LoVo grown in 2D (*N* = 3 biological replicates). **(C, D)** Full uncropped western blot of full-length MUC2 protein (540 kDa) (**C**) and β-actin (43 kDa) **(D)** in three different cell lines LS-174T, HT-29, and LoVo grown in 3D (*N* = 3 biological replicates). The blot was stripped after MUC2 staining, and residual MUC2 is seen in the staining of β-actin. **(E, F)** Full uncropped western blot of full-length MUC2 protein (540 kDa) **(E)** and β-actin (43 kDa) **(F)** in HT-29 CTRL and HT-29 MUC2 K.O. grown in 2D (*N* = 3 biological replicates). (**G**, **H**) Full uncropped western blot of full-length MUC2 protein (540 kDa) **(G)** and β-actin (43 kDa) **(H)** in different cell lines HT-29 CTRL, HT-29 MUC2 K.O. grown in 3D (*N* = 3 biological replicates). **(I, J)** Full uncropped western blot of full-length MUC2 protein (540 kDa) **(I)** and β-actin (43 kDa) **(J)** in different cell lines LS-174T CTRL, LS-174T MUC2 K.O grown in 2D (*N* = 3 biological replicates). **(K, L)** Full uncropped western blot of full-length MUC2 protein (540 kDa) **(K)** and β-actin (43 kDa) **(L)** in different cell lines LS-174T CTRL, LS-174T MUC2 K.O grown in 3D (*N* = 3 biological replicates).

Supplementary Figure 6
**(A)** Ice analysis results on bulk HT-29 using gRNA2. **(B)** Ice analysis results of HT-29 clone 22 using gRNA2. **(C)** Ice analysis results on bulk LS-174T using gRNA3. **(B)** Ice analysis results of HT-29 clone 11 using gRNA3.

Supplementary Figure 7
**(A)** Surface area analysis of HT-29 CTRL and HT-29 MUC2 K.O. spheroids on day 5 (*N* = 12 biological replicates). **(B)** Surface area analysis of LS-174T CTRL and HT-29 MUC2 K.O. spheroids on day 5 (*N* = 12 biological replicates).

Supplementary Figure 8
**(A)** Box plot of adherence junction enrichment score (ES) in HT-29 and LS-174T. The ES is upregulated in HT-29 MUC2 K.O. compared to CTRL (*p* = 0.041) (Student’s *t*-test). In contrast, the ES is downregulated in LS-174T MUC2 K.O. compared to CTRL (*p* = 0.047) (Student’s *t*-test).

Supplementary Figure 9
**(A)** Representative micrographs of one of the individual experiments at time points 0, 24, 48, and 72 h in HT-29 CTRL and HT-29 MUC2 K.O. No cell motility toward the gap area was seen even after 72 h despite a clear cell multiplication. **(B)** Size of scratch in HT-29 CTRL and MUC2 K.O. relative to 0 h (*N* = 12). No statistical difference was measured between CTRL and MUC2 K.O. at any timepoint. **(C)** Representative micrographs of one of the individual experiments at time points 0, 24, 48, and 72 h in LS-174T CTRL and LS-174T MUC2 K.O. No cell motility toward the gap area was seen even after 72 h despite a clear cell multiplication. **(D)** Size of scratch in LS-174T CTRL and MUC2 K.O. relative to 0 h (*N* = 16). No statistical difference was measured between CTRL and MUC2 K.O. at any timepoint.

Supplementary Figure 10
**(A)** Representative capillary western blot (CWB) results of E-cadherin, N-cadherin, and vimentin expression in human fetal fibroblast (HFF), Hela, HT-29 CTRL, and HT-29 MUC2 K.O. (*N* = 2 technical replicates). **(B)** Representative capillary western blot (CWB) results of E-cadherin, N-cadherin, and vimentin expression in HFF, Hela, LS-174T CTRL, and LS-174T MUC2 K.O. (*N* = 2 technical replicates). **(C)** Quantification of band intensity of E-cadherin, N-cadherin, and vimentin normalized to β-actin expression in HFF, Hela, HT-29 CTRL, and HT-29 MUC2 (*N* = 2, technical replicates). **(D)** Quantification of band intensity of E-cadherin, N-cadherin, and vimentin normalized to β-actin expression in HFF, Hela, LS-174T CTRL, and LS-174T MUC2 K.O. (*N* = 2, technical replicates).

Supplementary Figure 11 Comparison of epithelial mesenchymal transition enrichment score between CTRL (2D) and MUC2 K.O. (2D) and between CTRL (3D) and MUC2 K.O. (3D) in **(A)** HT-29 and **(B)** LS-174T. *P*-value from Student’s *t*-test.

Supplementary Figure 12
**(A)** Average distance covered in micrometer per minute by each HT-29 and LS-174T CTRL and MUC2 K.O. counterpart. One-way ANOVA shows that no statistical difference was observed between CTRL and MUC2 K.O. in either cell line, yet LS-174T CTRL has a slightly higher motility than HT-29 CTRL (*p* = 0.0012), and LS-174T MUC2 K.O. also has a higher motility than HT-29 MUC2 K.O.(*p* = 0.0154). **(B)** Average velocity in micrometer per minute of each HT-29 and LS-174T CTRL and MUC2 K.O. counterpart. Again, one-way ANOVA shows that no statistical difference was observed between CTRL and MUC2 K.O. in either cell lines, but LS-174T CTRL has a slightly higher velocity than HT-29 CTRL (*p* = 0.0012), and LS-174T MUC2 K.O. also has greater velocity than HT-29 MUC2 K.O. (*p* = 0.0154). **(C)** Example of video of HT-29 CTRL over 40 h used for the analysis of motility and velocity. **(D)** Example of video of HT-29 MUC2 K.O. over 40 h used for the analysis of motility and velocity. **(E)** Example of video of LS-174T CTRL over 40 h used for the analysis of motility and velocity. **(F)** Example of video of LS-174T MUC2 K.O. over 40 h used for the analysis of motility and velocity.

Supplementary Figure 13
**(A)** Number of colonies formed in 8 days for each HT-29 cell type in a six-well plate (*N* = 6 technical replicates). Unpaired *t*-test reveals no significant difference between HT-29 CTRL and HT-29 MUC2 K.O. **(B)** Number of colonies formed in 8 days for each LS-174T cell type in a six-well plate (*N* = 6 technical replicates). Unpaired *t*-test reveals no significant difference between LS-174T CTRL and LS-174T MUC2 K.O.

Supplementary Figure 14
**(A)** HT-29 CTRL and MUC2 K.O. growth and death monitored by GFP expression over 7 days in 2D setup. The top panel shows the GFP reading for HT-29 CTRL only with 0 to 10,000 E/A PBMCs added on day 2. The following panel represents the comparison of HT-29 CTRL and HT-29 MUC2 K.O. with a defined number of E/A PBCMs. Data are presented as relative to the same cell line without co-culture with PBMCs (*N* = 18, three biological replicates of six technical replicates). **(B)** LS-174T CTRL and MUC2 K.O. growth and death monitored by GFP expression over 7 days in 2D setup. The top panel shows the GFP reading for LS-174T CTRL only with 0 to 10,000 E/A PBMCs added on day 2. The following panel represents the comparison of LS-174T CTRL and LS-174T MUC2 K.O. with a defined number of E/A PBCMs. Data are presented as relative to the same cell line without co-culture with PBMCs (*N* = 18, three biological replicates of six technical replicates). **(C)** HT-29 CTRL and MUC2 K.O. growth and death monitored by GFP expression over 9 days in 3D setup. The top panel shows the GFP reading for HT-29 CTRL only with 0 to 10,000 E/A PBMCs added on day 5. The following panel represents the comparison of HT-29 CTRL and HT-29 MUC2 K.O. with a defined number of E/A PBCMs. Data are presented as relative to the same cell line without co-culture with PBMCs (*N* = 18, three biological replicates of six technical replicates). **(D)** LS-174T CTRL and MUC2 K.O. growth and death monitored by GFP expression over 7 days in 3D setup. The top panel shows the GFP reading for LS-174T CTRL only with 0 to 10,000 E/A PBMCs added on day 5. The following panel represents the comparison of LS-174T CTRL and LS-174T MUC2 K.O. with a defined number of E/A PBCMs. Data are presented as relative to the same cell line without co-culture with PBMCs (*N* = 18, three biological replicates of six technical replicates). **(E)** Comparison of HT-29 with LS-174T CTRL or MUC2 K.O. with various numbers of E/A PBMCs in 2D. Data are presented as relative to the same cell line without co-culture with PBMCs (0 E/A PBMCs) (*N* = 18, three biological replicates of six technical replicates). **(F)** Comparison of HT-29 with LS-174T CTRL or MUC2 K.O. with various numbers of E/A PBMCs in 3D. Data are presented as relative to the same cell line without co-culture with PBMCs (0 E/A PBMCs) (*N* = 18, three biological replicates of six technical replicates).

Supplementary Figure 15 Representative video of HT-29 CTRL, HT-29 MUC2 K.O., LS-174T CTRL, and LS-174T MUC2 K.O. from days 5 to 7 with and without E/A PBMCs.

Supplementary Figure 16 Relative percentages of T cells (defined as CD45^+^ Epcam^-^ CD4^+^ or CD45^+^ Epcam^-^ CD8^+^ cells) and NK cells (defined as CD45^+^ Epcam^-^ CD56^+^ cells) in the IN and OUT fractions after co-culture with HT-29 and LS-174T CTRL or MUC2 K.O. cells. No specific enrichment or selection of either cell type was observed in the IN or OUT fractions compared to E/A PBMCs alone.

Supplementary Figure 17 IPA pathway enrichment analysis. Top 20 significant pathways based on *p*-value associated with DEG (FDR < 0.01, logFC ≥ 1) between MUC2 K.O. (2D) and CTRL (2D) in **(A)** HT-29 (*n* = 557) and **(C)** LS-174T (*n* = 1,636) and between MUC2 K.O (3D) and CTRL (3D) in **(B)** HT-29 (*n* = 345) and **(D)** LS-174T (*n* = 463). Histograms represent the proportion (%) of DEGs upregulated (red) or downregulated (green) in MUC2 K.O *versus* CTRL. The circles represent the pathway activation status. The blue circle indicates that the pathway is inhibited with a negative z-score, the orange circle represents the pathway that is activated with a positive z-score, the white circle represents the pathway that is neutral with zero z-score, while the gray circle indicates that the pathway activity is unknown.

Supplementary Figure 18 Comparison of ICR score mean value between CTRL + E/A PBMCs IN fraction and MUC2 K.O. + E/A PBMCs IN fraction in HT-29 and LS-174T. *P*-value from Student’s *t*-test.

Supplementary Figure 19 Comparison of gene expression of other gel-forming mucin in HT-29 and LS-174T spheroids between MUC2 K.O. (in blue) and CTRL (in black) (3D). Statistical analysis represented as *p*-values from Student’s *t*-test.

Supplementary Figure 20 Immunostaining of HT-29 CTRL **(A)**, HT-29 MUC2 K.O. **(B)**, LS-174T CTRL **(C)**, and LS-174T MUC2 K.O. **(D)** cells for MUC2, MUC5AC, and MUC5B, with negative controls using goat anti-rabbit (GAR) and goat anti-mouse (GAM) antibodies alone.

Supplementary Figure 21
**(A)** Western blot of full-length MUC5AC protein (641 kDa) and calnexin (housekeeping gene, 67 kDa) in HT-29 CTRL, HT-29 MUC2 K.O., LS-174T CTRL, and LS-174T MUC K.O. **(C, D)** Full uncropped western blot of full-length MUC5AC protein (641 kDa) **(A)** and calnexin (housekeeping gene, 67 kDa) **(B)** in HT-29 CTRL, HT-29 MUC2 K.O., LS-174T CTRL, and LS-174T MUC K.O. grown in 2D (*N* = 1 biological replicate).
