# Supplementary material for: MUC2 expression modulates immune infiltration in colorectal cancer
Source: Front Immunol. 2025 Jan 24;15:1500374. doi: 10.3389/fimmu.2024.1500374 (PMC11802499; doi:10.3389/fimmu.2024.1500374)

A

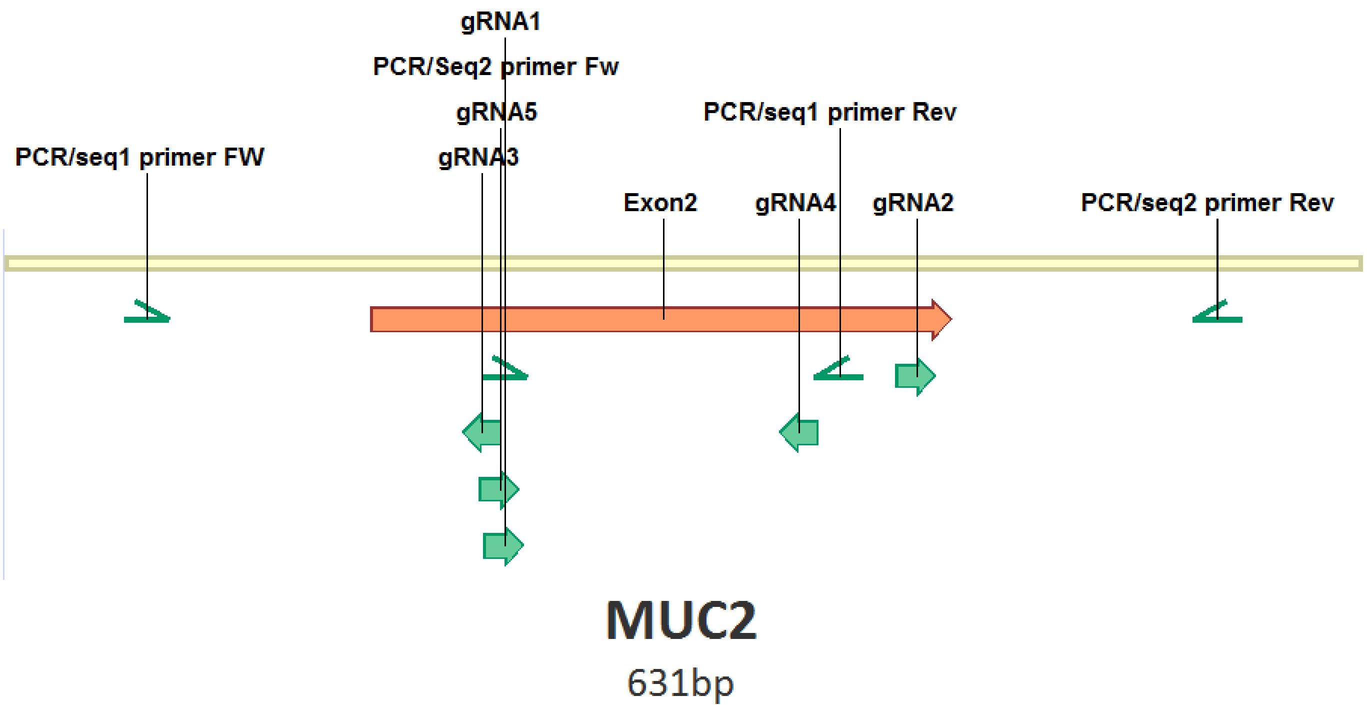
gRNA1
PCR/Seq2 primer Fw
gRNA5
PCR/seq1 primer Rev
PCR/seq1 primer FW
gRNA3
Exon2
gRNA4
gRNA2
PCR/seq2 primer Rev
MUC2
631bp

Supplementary figure 2

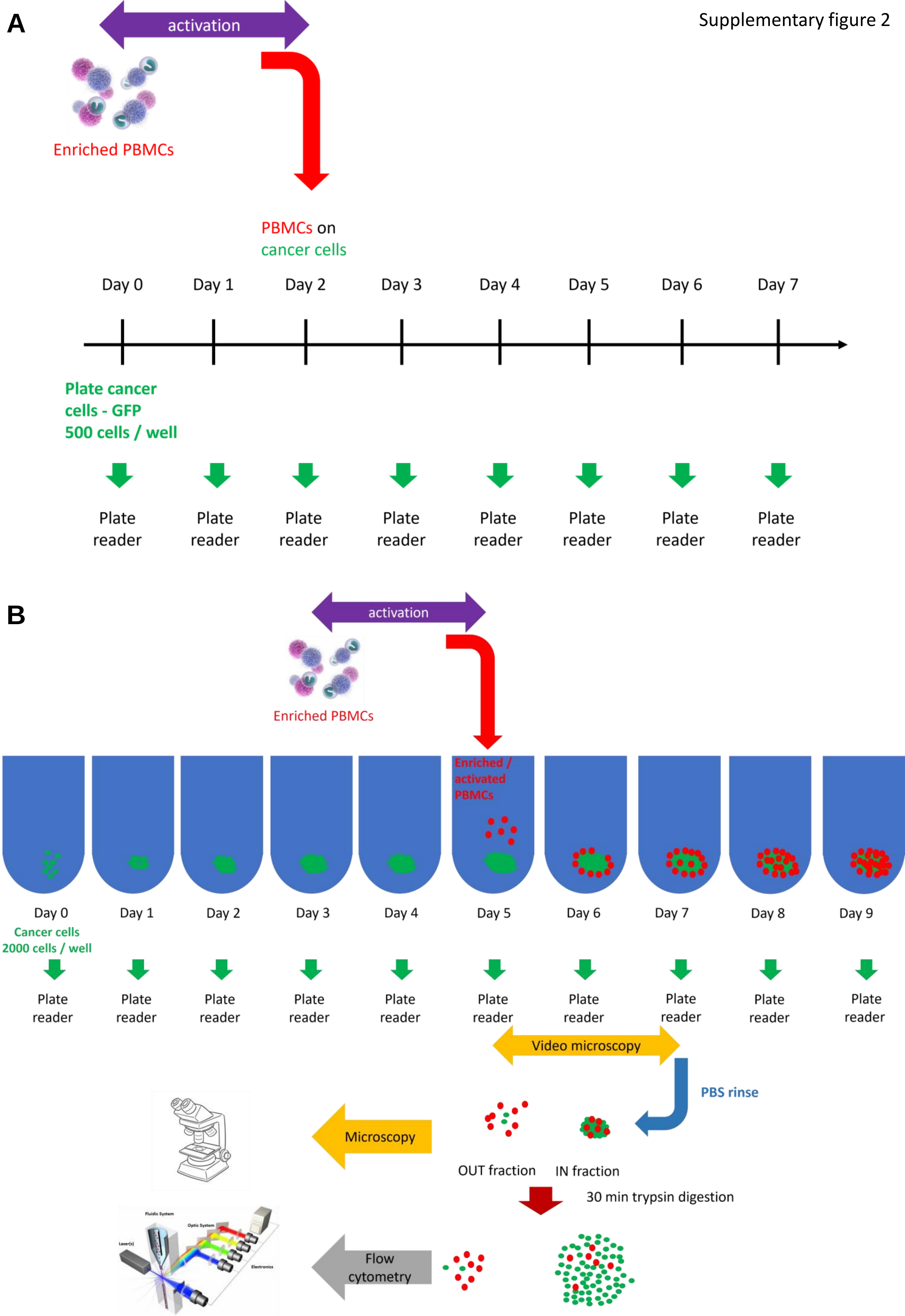

Supplementary figure 3

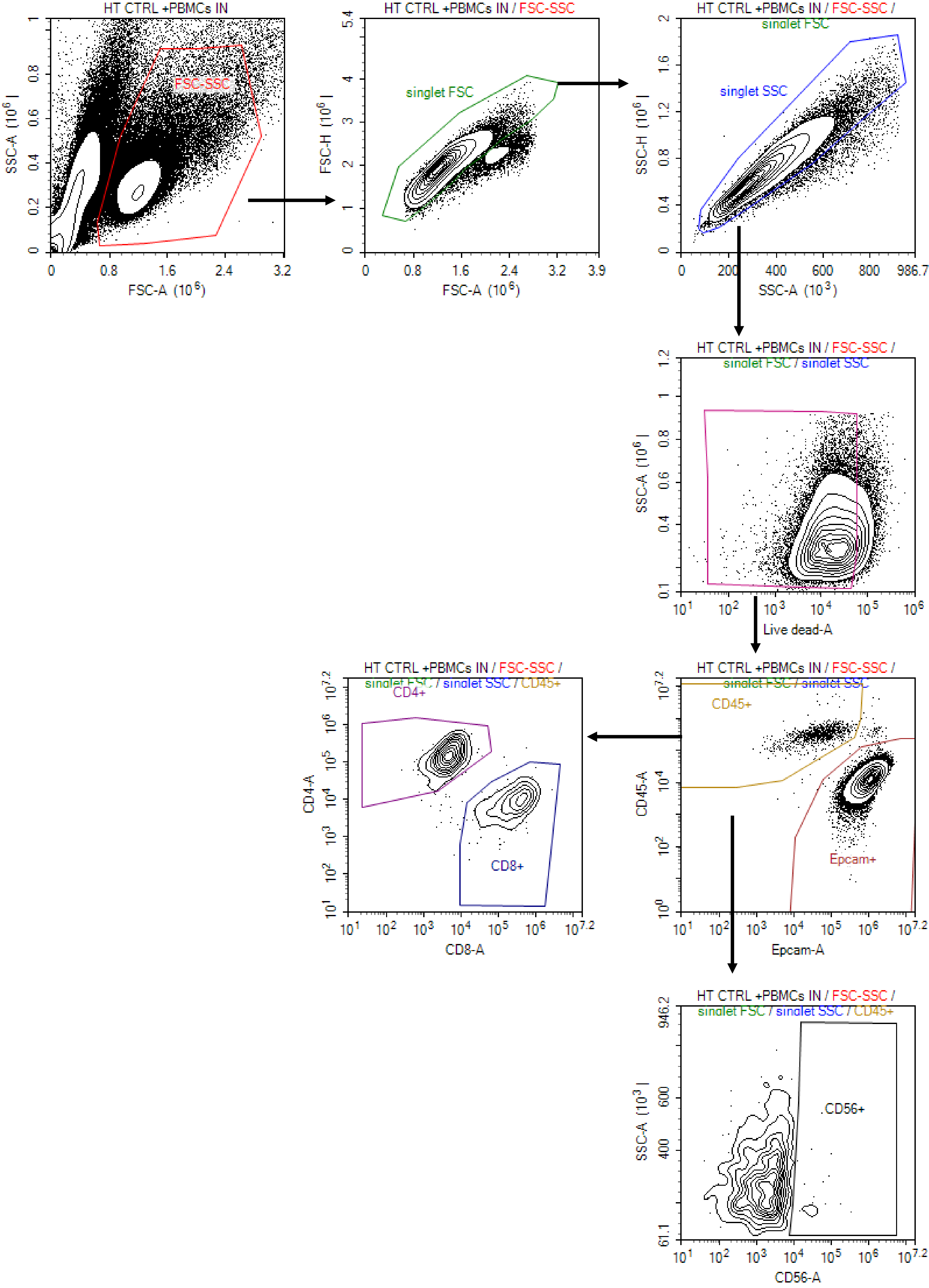

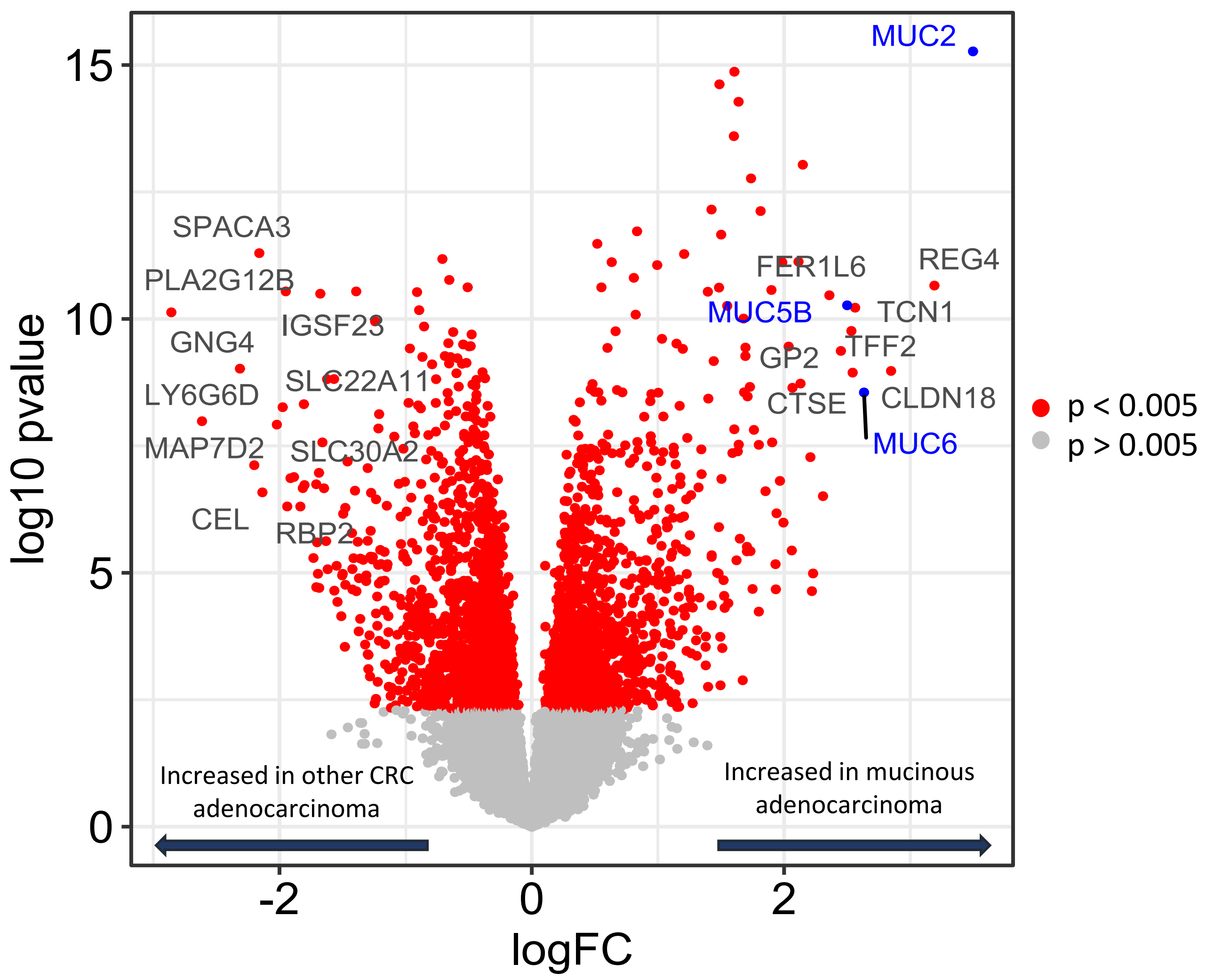
MUC2
SPACA3
PLA2G12B
IGSF23
GNG4
SLC22A11
LY6G6D
MAP7D2
SLC30A2
CEL
RBP2
FER1L6
REG4
MUC5B
TCN1
TFF2
GP2
CTSE
CLDN18
MUC6
15
10
5
0
-2
0
2
log10 pvalue
logFC
p < 0.005
p > 0.005
Increased in other CRC adenocarcinoma
Increased in mucinous adenocarcinoma

Supplementary figure 5

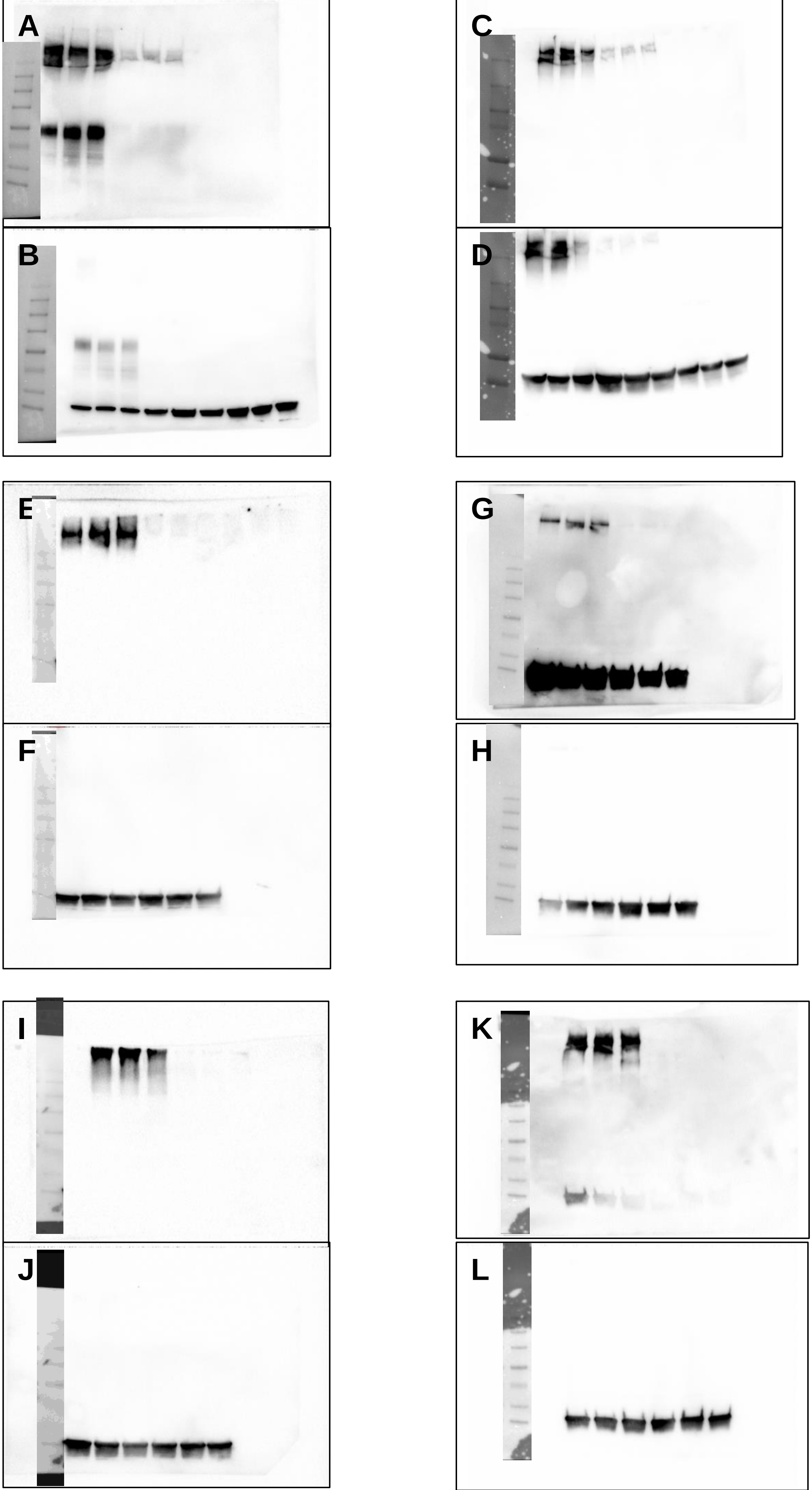

Supplementary figure 6

A

Status	Guide Target	PAM Sequence	Indel %	Model Fit (R^2)	Knockout-Score
Succeeded	CCACCTGGCTGTGCTTAACG	GGG	55	0.93	46

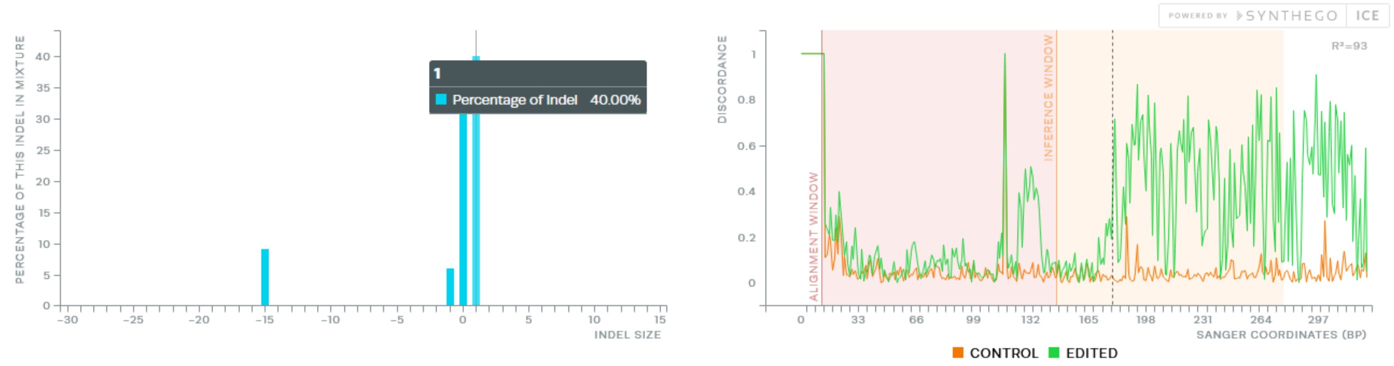

B

Status	Guide Target	PAM Sequence	Indel %	Model Fit (R^2)	Knockout-Score
Succeeded	CCACCTGGCTGTGCTTAACG	GGG	98	0.98	98

POWERED BY SYNTHEGO ICE

RELATIVE CONTRIBUTION OF EACH SEQUENCE (NORMALIZED)

INDEL	CONTRIBUTION	SEQUENCE
+2	98%	CTCACCCGCCACCTGGCTGTGCTTA : NNACGGGGCCGTGTGAGTGTGGTCGGTGGCACCCCTCCCACATCCTAGCA

C

Status	Guide Target	PAM Sequence	Indel %	Model Fit (R^2)	Knockout-Score
Succeeded	CTTGTAGTGGAAGTTGCCCC	AGG	57	0.86	51

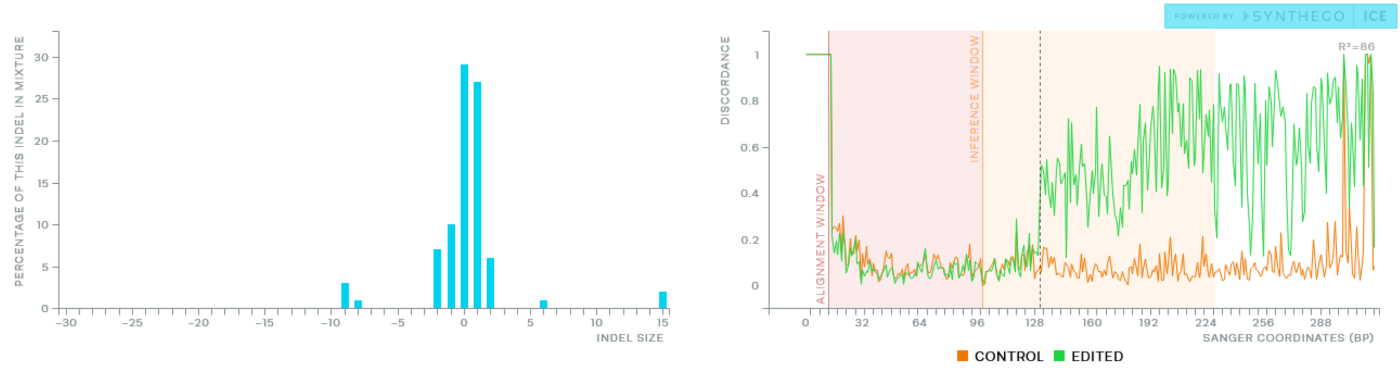

D

Status	Guide Target	PAM Sequence	Indel %	Model Fit (R^2)	Knockout-Score
Succeeded	CTTGTAGTGGAAGTTGCCCC	AGG	93	0.93	93

POWERED BY SYNTHEGO ICE

RELATIVE CONTRIBUTION OF EACH SEQUENCE (NORMALIZED)

INDEL	CONTRIBUTION	SEQUENCE
+2	49%	TCGAAGGTCTTGTAGTGGAAGTTGC : NNCCCAGGTGCTGCAGACGTTGTGGCCGTGGTTTCGGGTTCTGCCCTCTG
−2	43%	TCGAAGGTCTTGTAGTGGAAGTTGC : --CAGGTGCTGCAGACGTTGTGGCCGTGGTTTCGGGTTCTGCCCTCTGAG
−2	1%	TCGAAGGTCTTGTAGTGGAAGTTG- : -CCAGGTGCTGCAGACGTTGTGGCCGTGGTTTCGGGTTCTGCCCTCTGAG

Supplementary figure 7

A

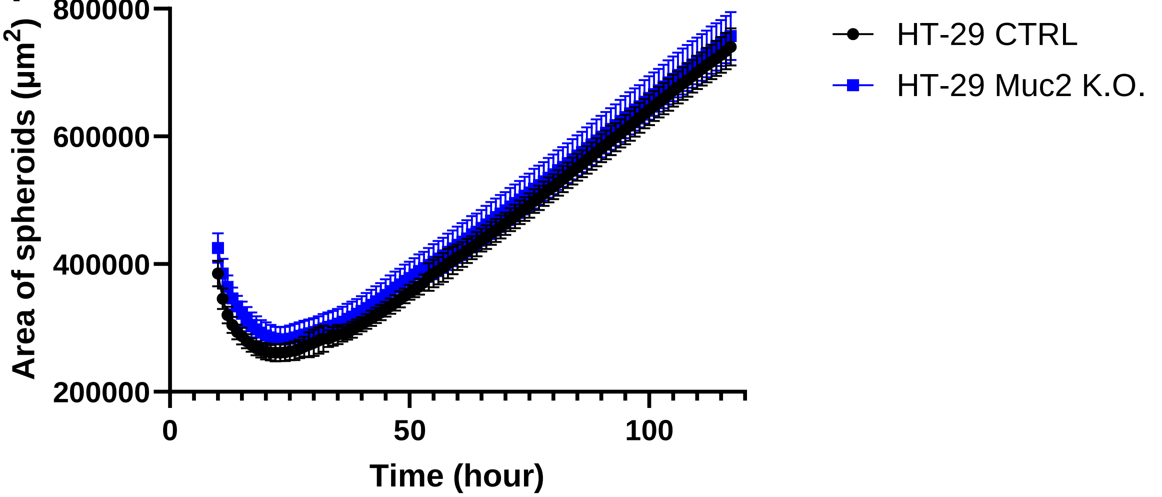

B

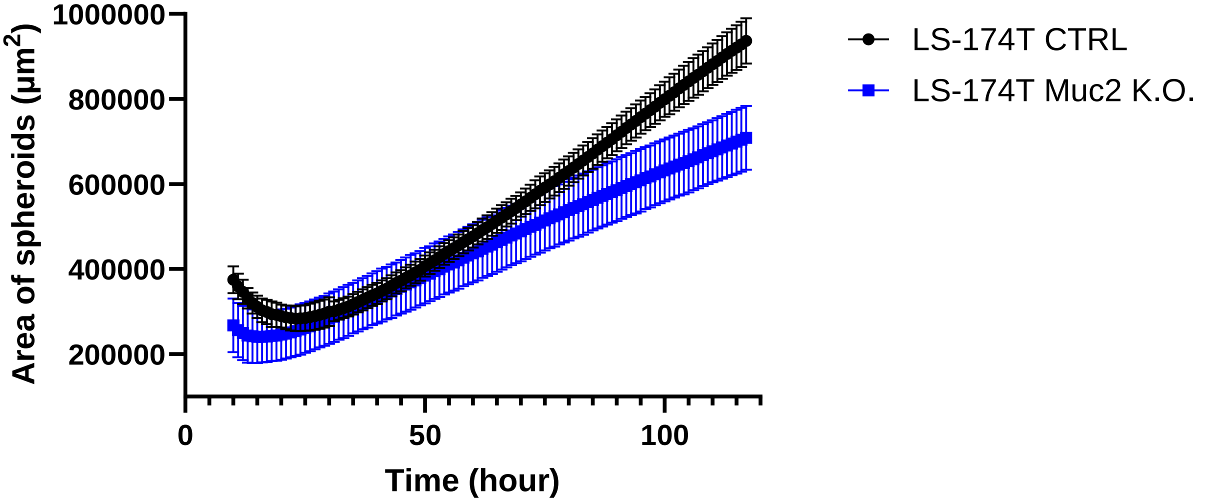

A

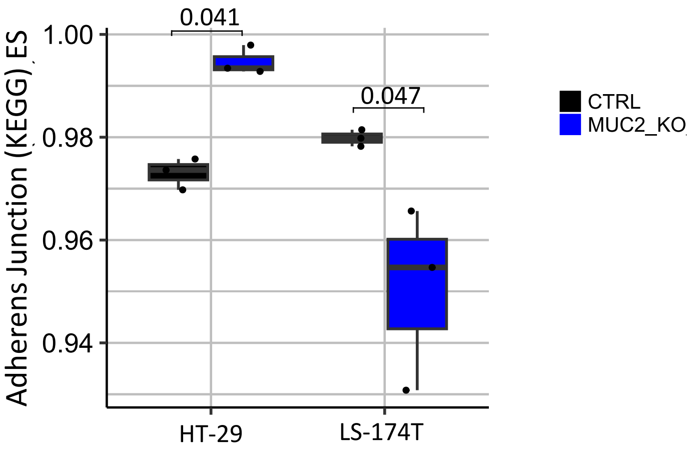
0.041
0.047
1.00
0.98
0.96
0.94
Adherens Junction (KEGG) ES
HT-29
LS-174T
CTRL
MUC2_KO

Supplementary figure 9

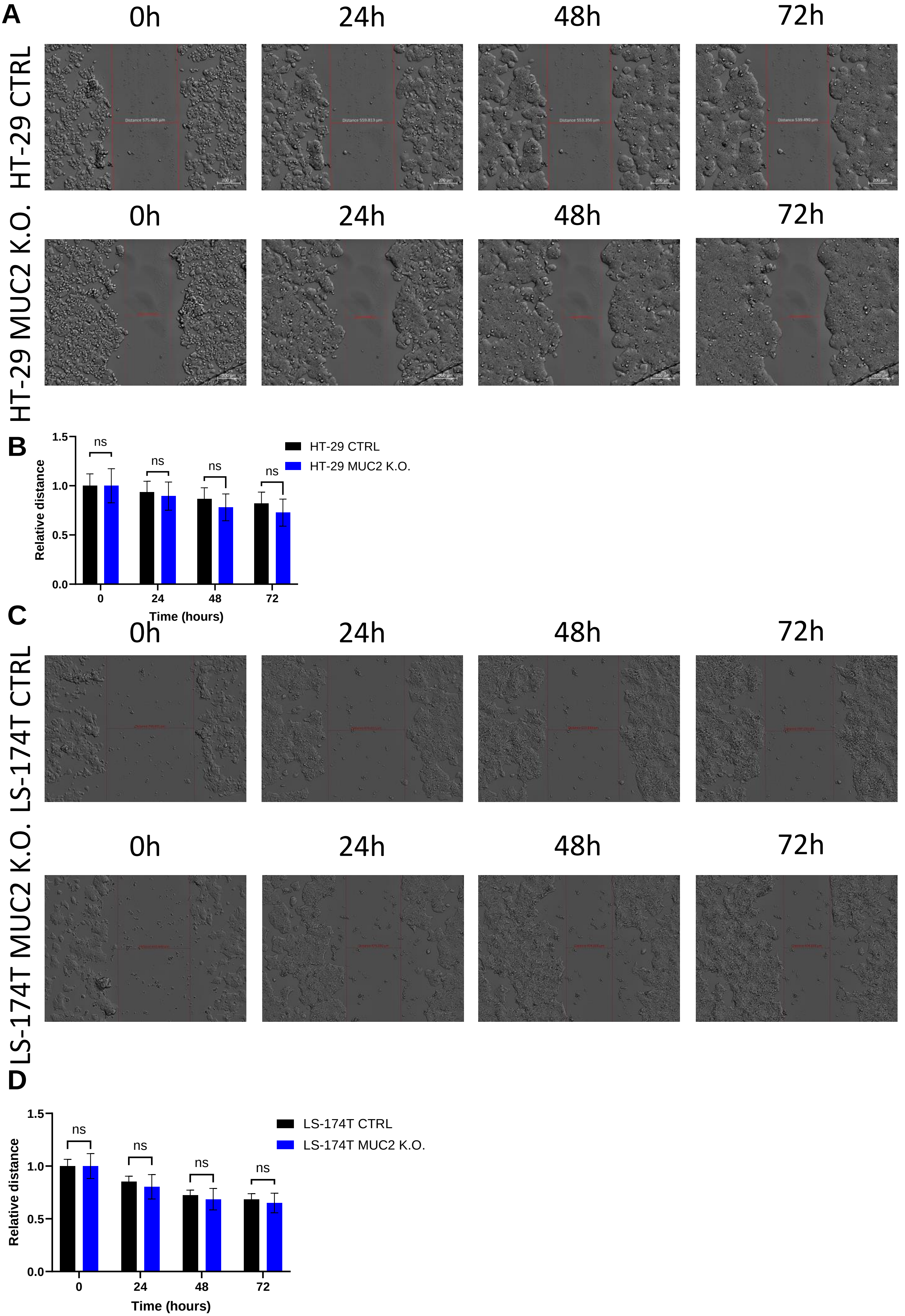

Supplementary figure 10

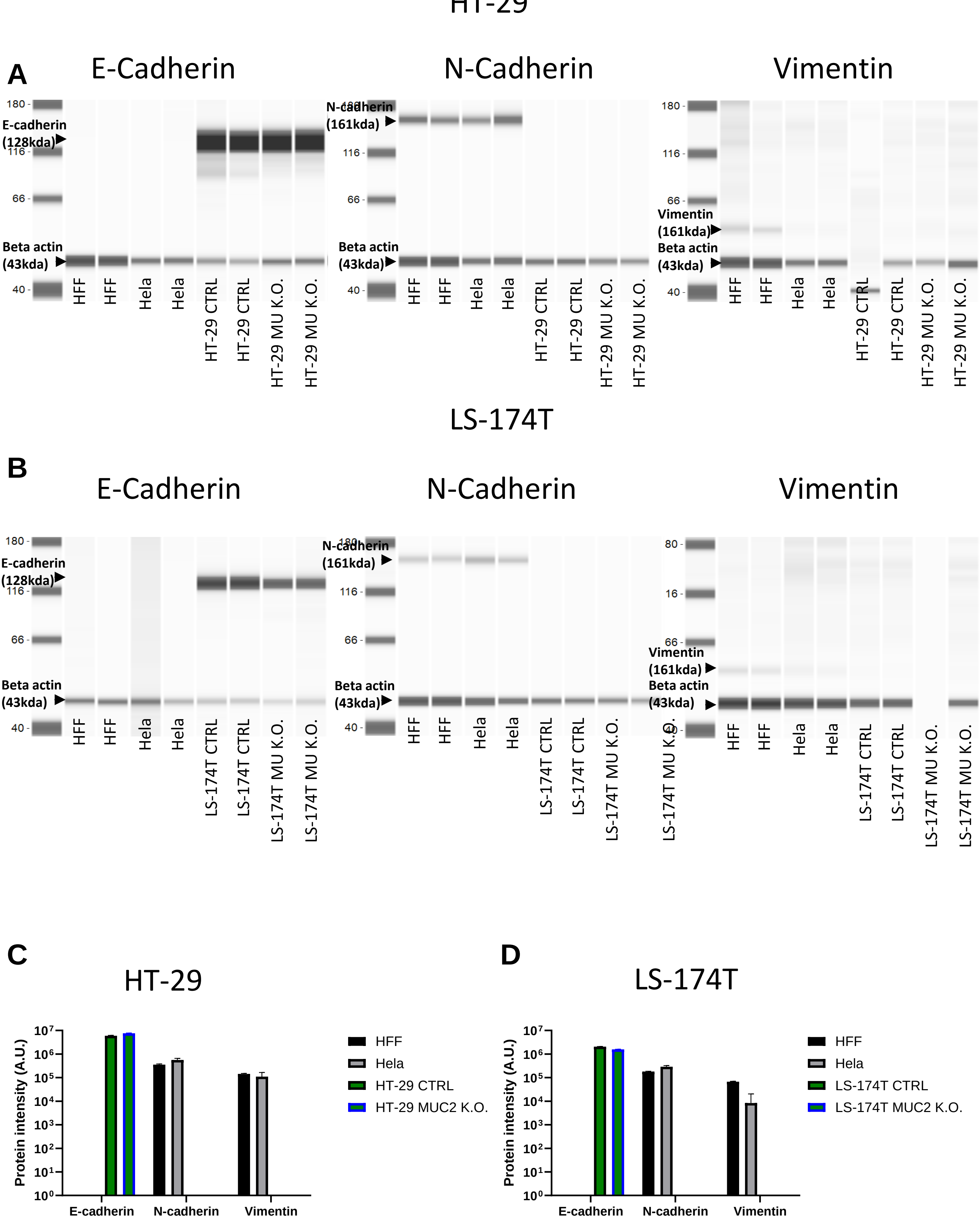

A

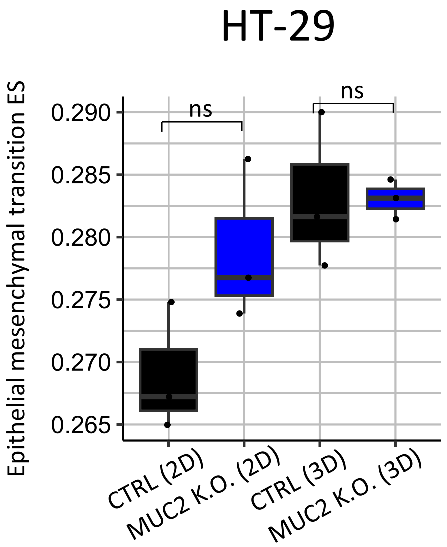
HT-29
Epithelial mesenchymal transition ES
0.290
0.285
0.280
0.275
0.270
0.265
ns
ns
CTRL (2D)
MUC2 K.O. (2D)
CTRL (3D)
MUC2 K.O. (3D)

B

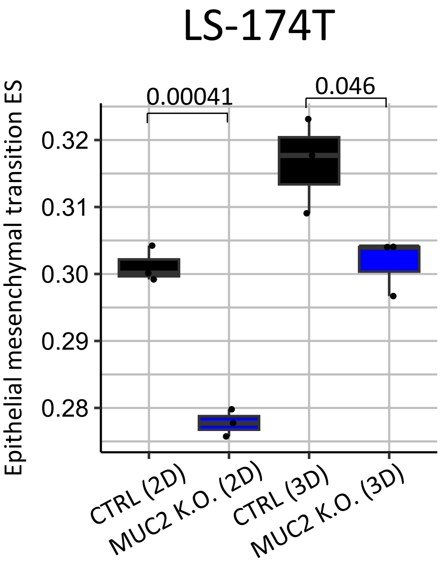
LS-174T
Epithelial mesenchymal transition ES
0.32
0.31
0.30
0.29
0.28
0.00041
0.046
CTRL (2D)
MUC2 K.O. (2D)
CTRL (3D)
MUC2 K.O. (3D)

Supplementary figure 12

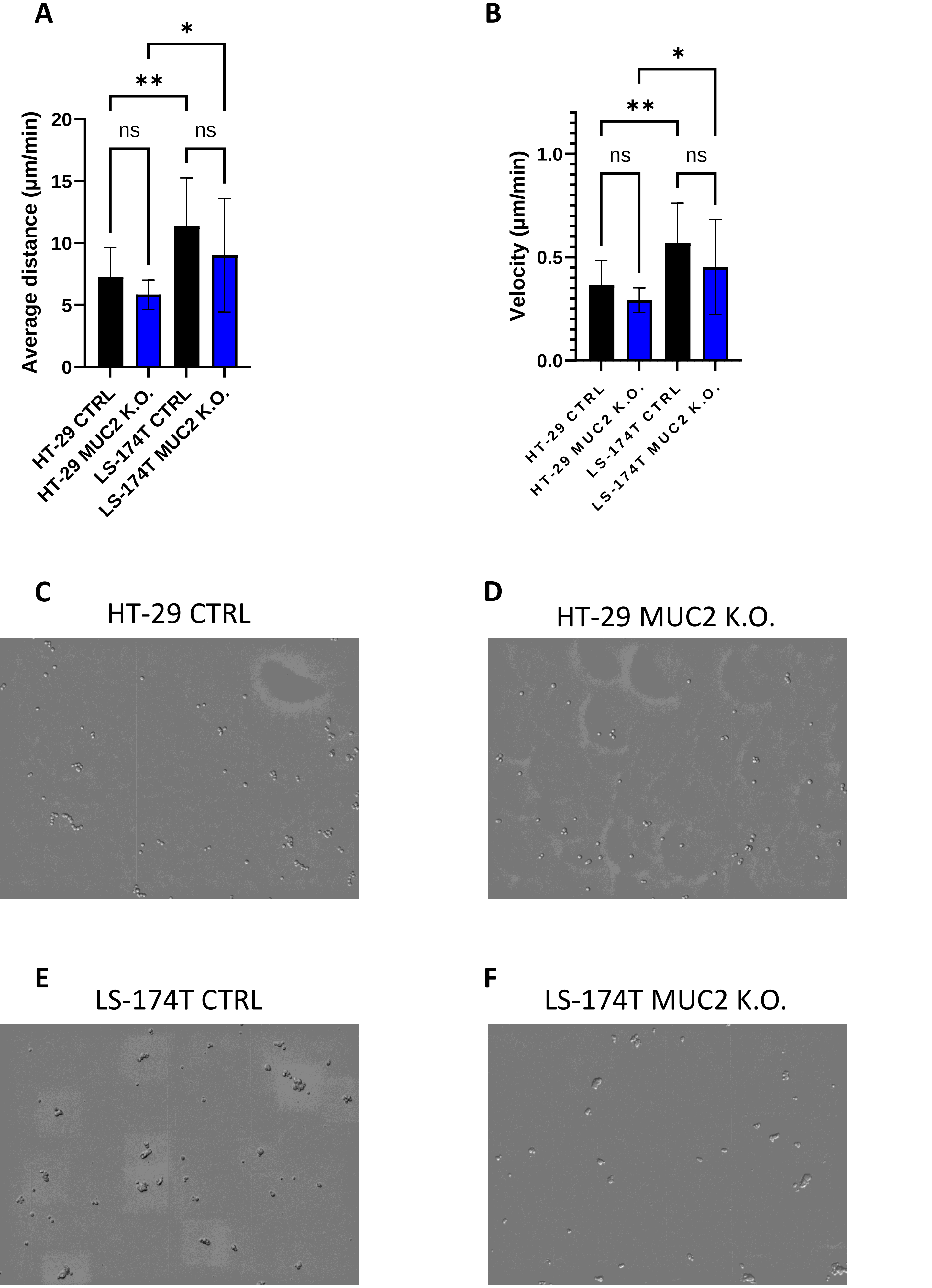

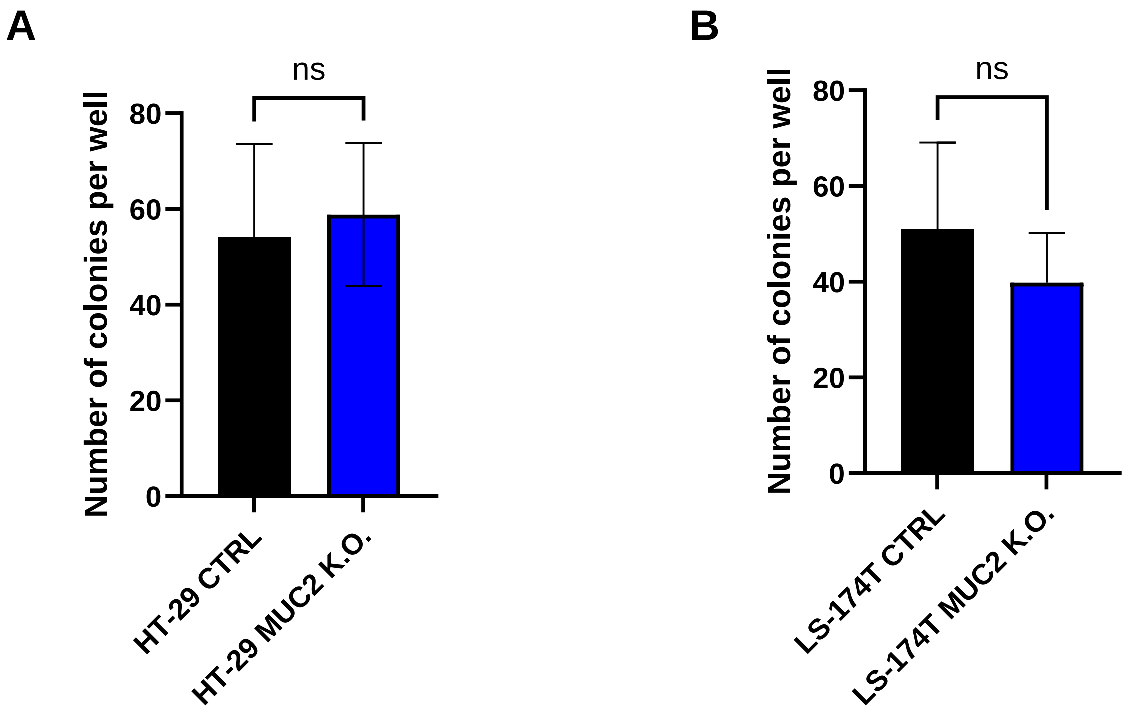
A
ns
Number of colonies per well
80
60
40
20
0
HT-29 CTRL
HT-29 MUC2 K.O.
B
ns
Number of colonies per well
80
60
40
20
0
LS-174T CTRL
LS-174T MUC2 K.O.

Supplementary figure 14

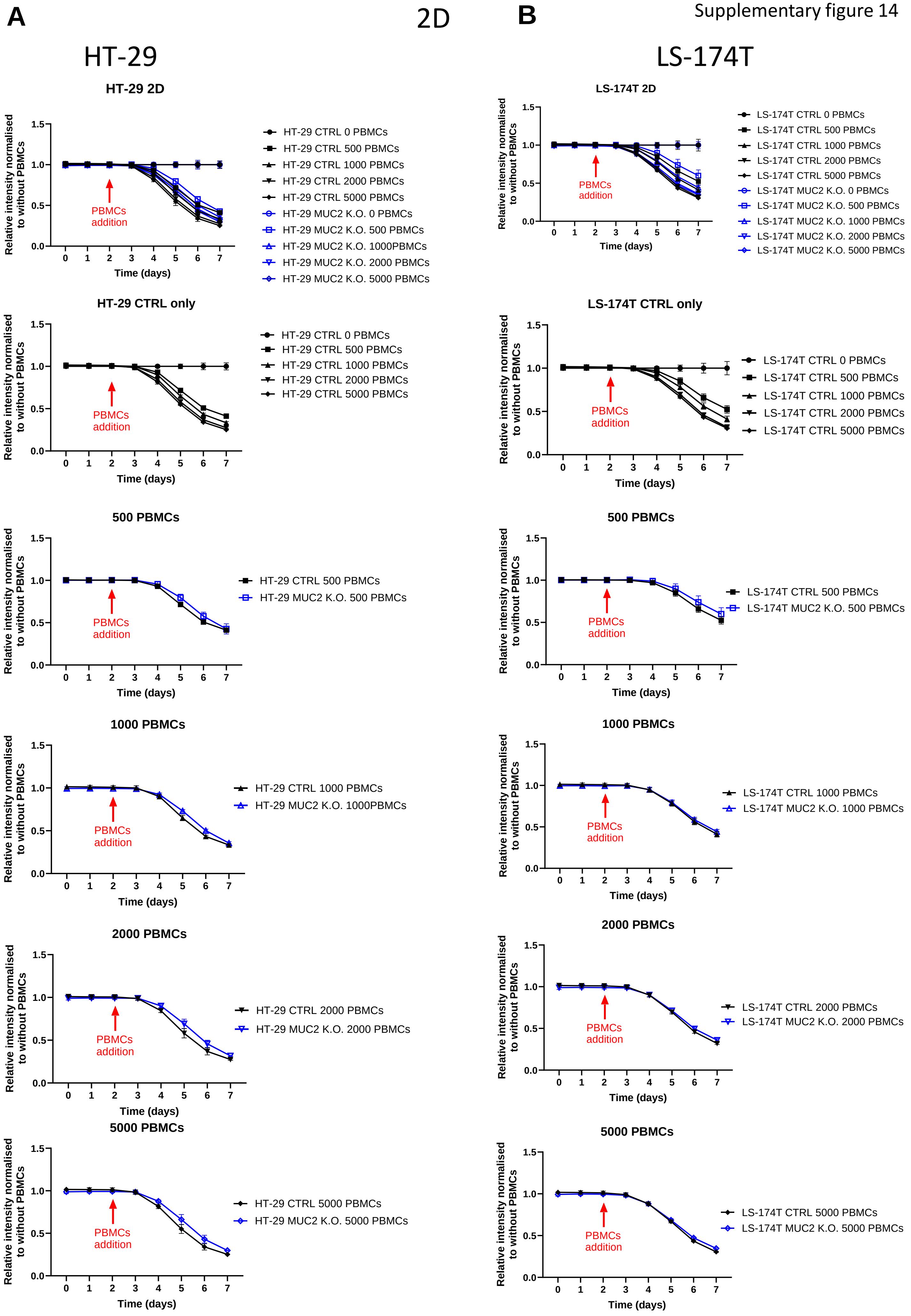

C   3D   D

Supplementary figure 14

HT-29

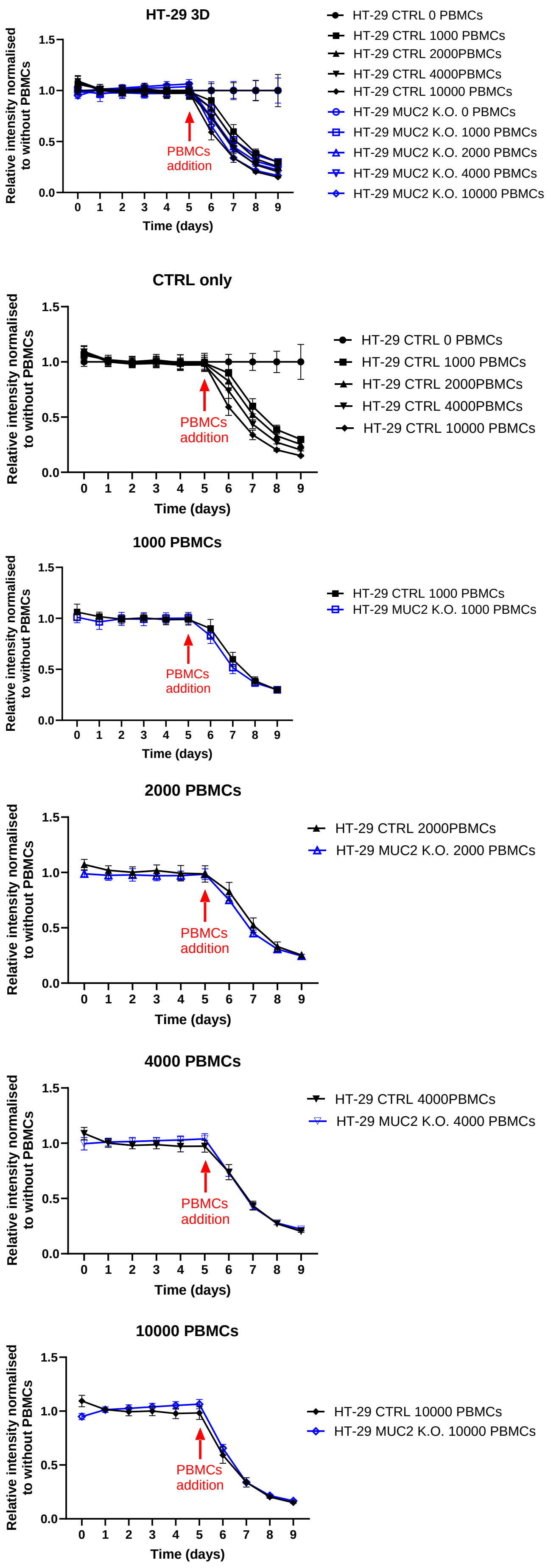

LS-174T

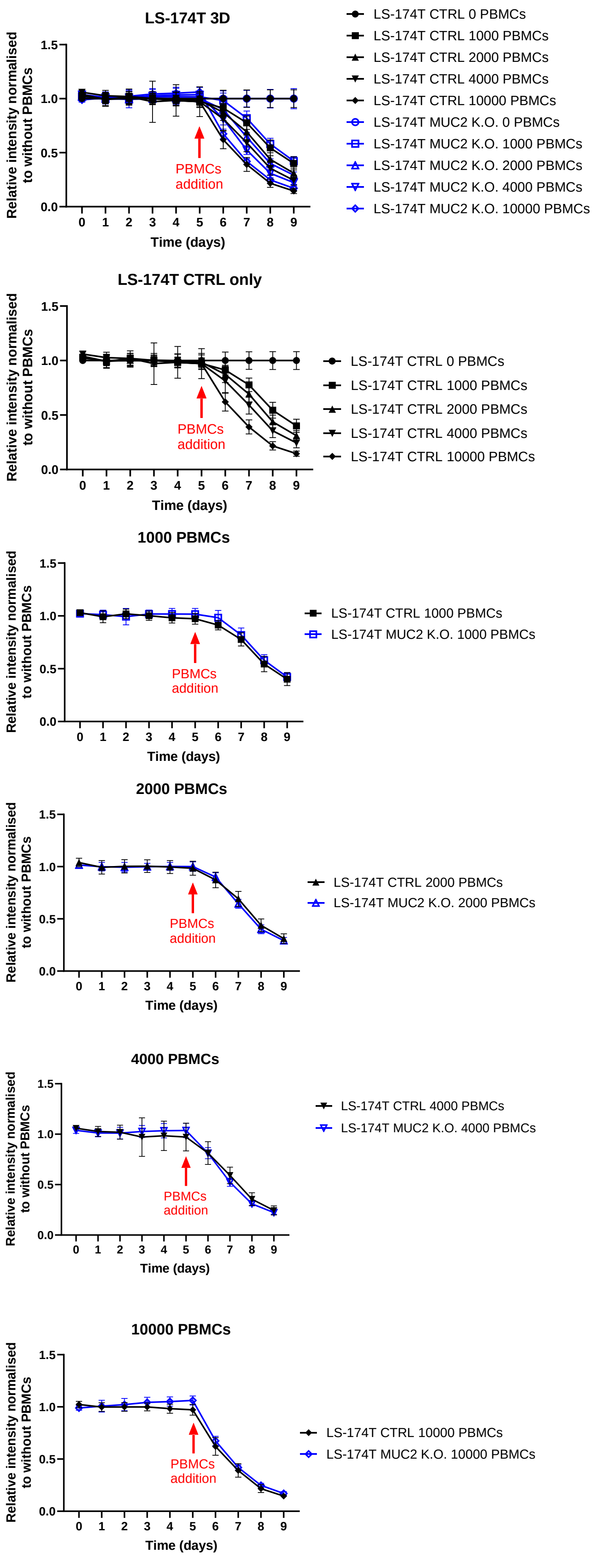

Supplementary figure 14

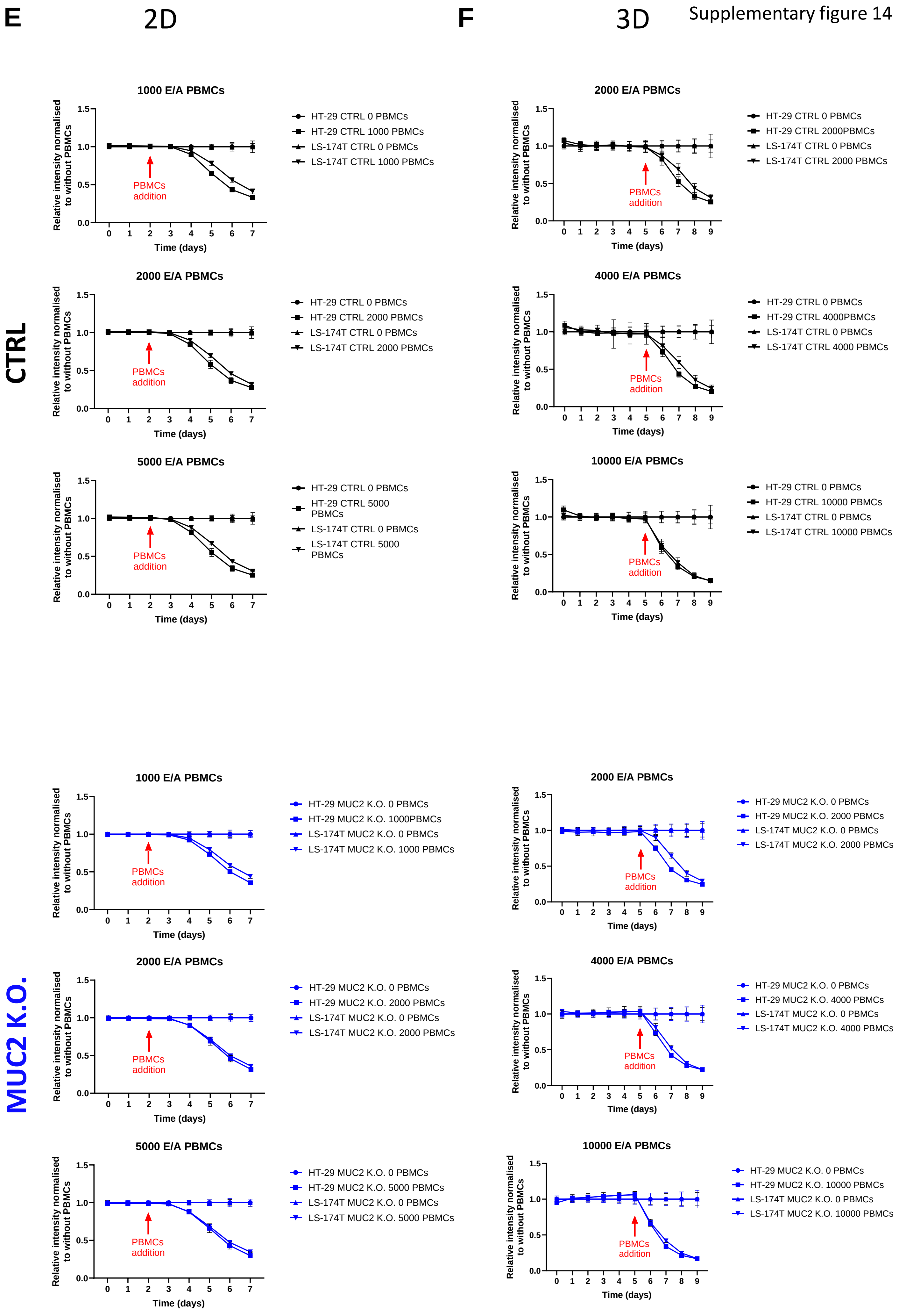

Supplementary figure 15

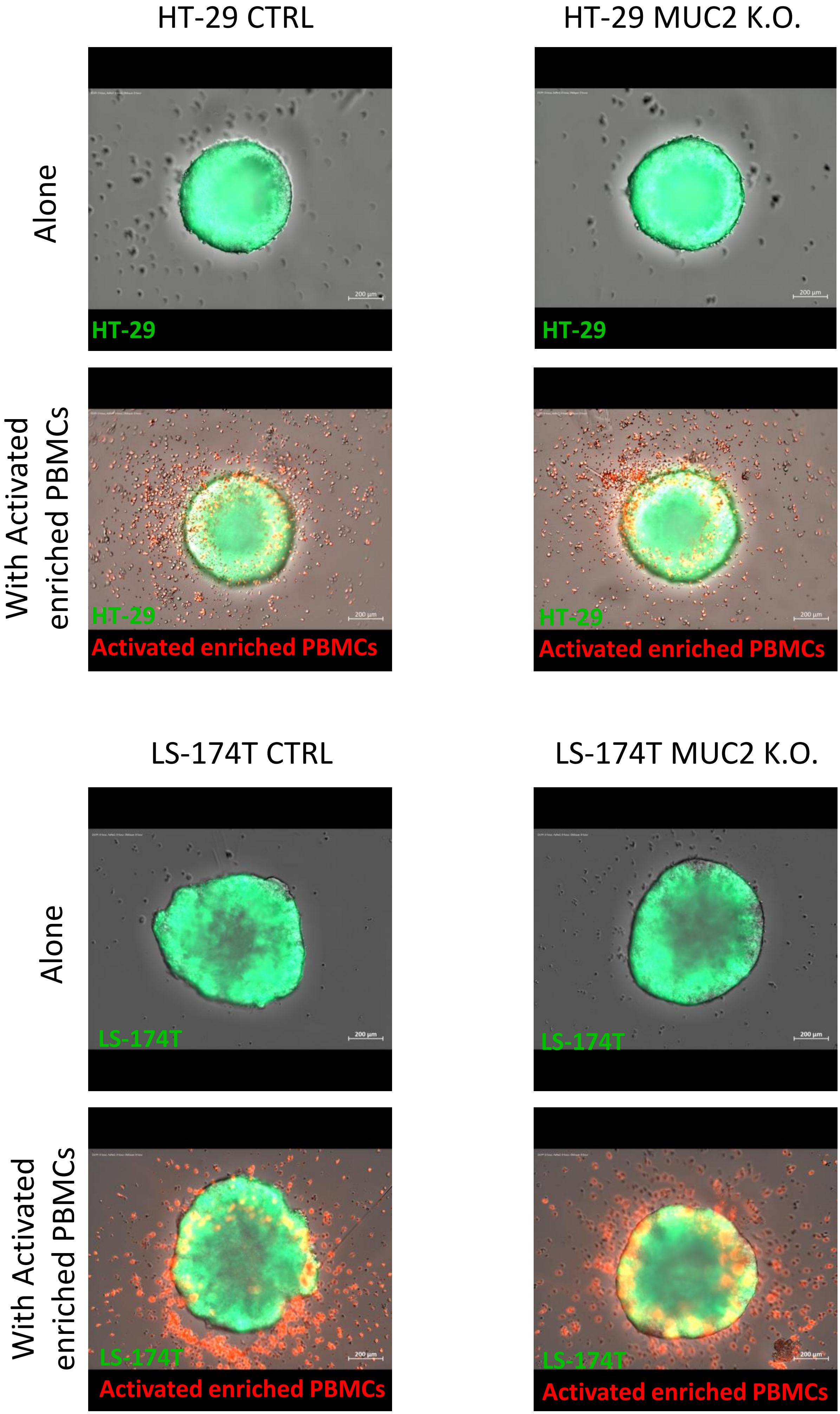

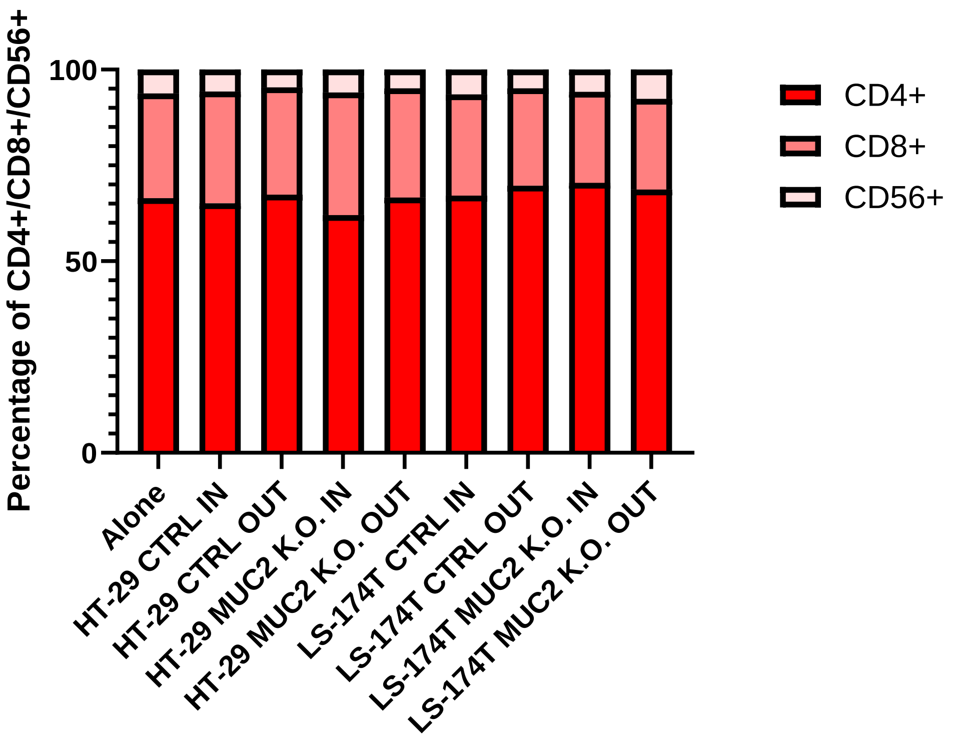

Percentage of CD4+/CD8+/CD56+
100
50
0
Alone
HT-29 CTRL IN
HT-29 CTRL OUT
HT-29 MUC2 K.O. IN
HT-29 MUC2 K.O. OUT
LS-174T CTRL IN
LS-174T CTRL OUT
LS-174T MUC2 K.O. IN
LS-174T MUC2 K.O. OUT
CD4+
CD8+
CD56+

A

HT-29

DEG

MUC2 K.O. (2D) vs. CTRL (2D)

FDR < 0.01, logFC >=1

Pathways cutoff: Top 20 pathways

Total: 557 DEG

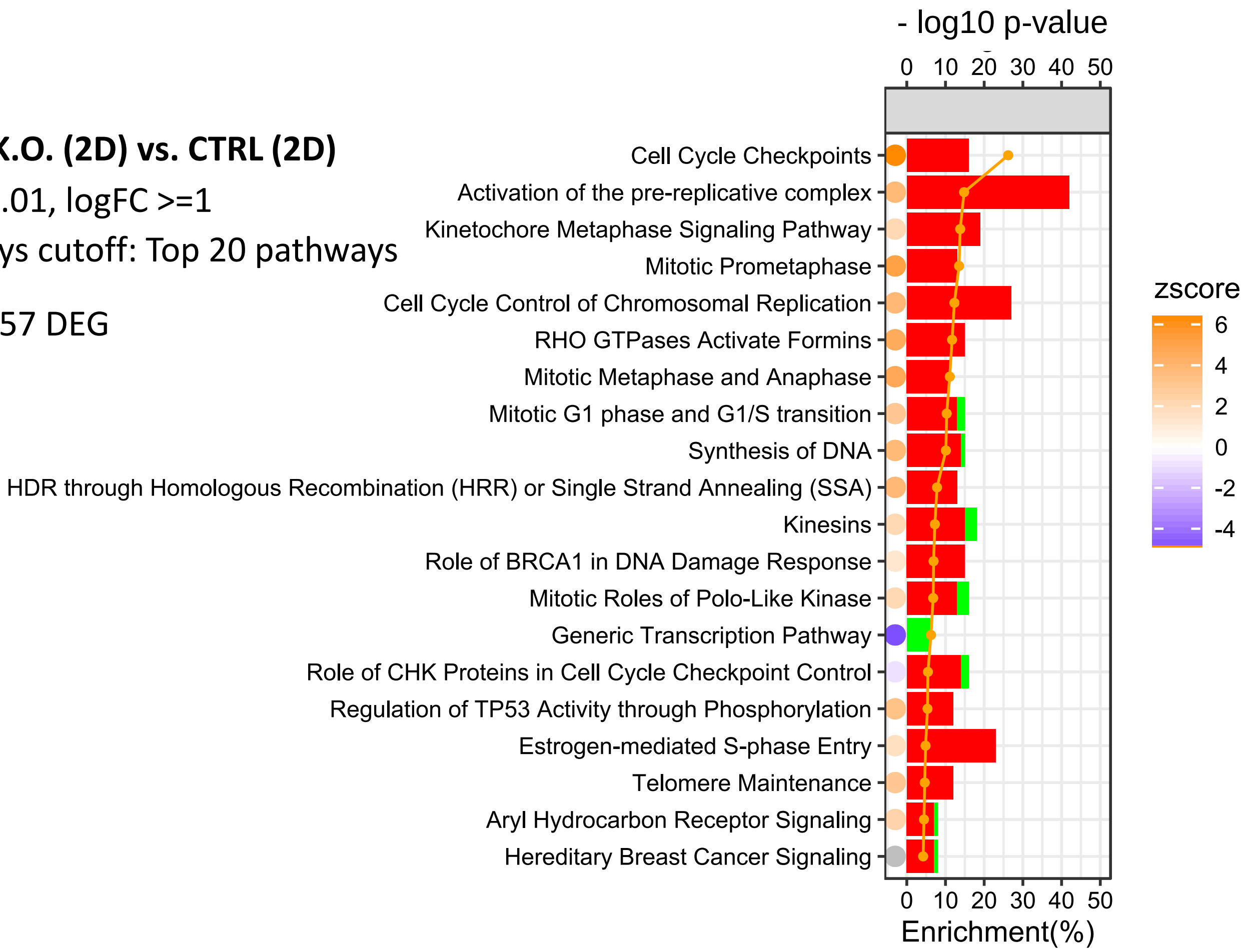

B

MUC2 KO (3D) vs. CTRL (3D)

FDR < 0.01, logFC >=1

Pathways cutoff: Top 20 pathways

Total: 345 DEG

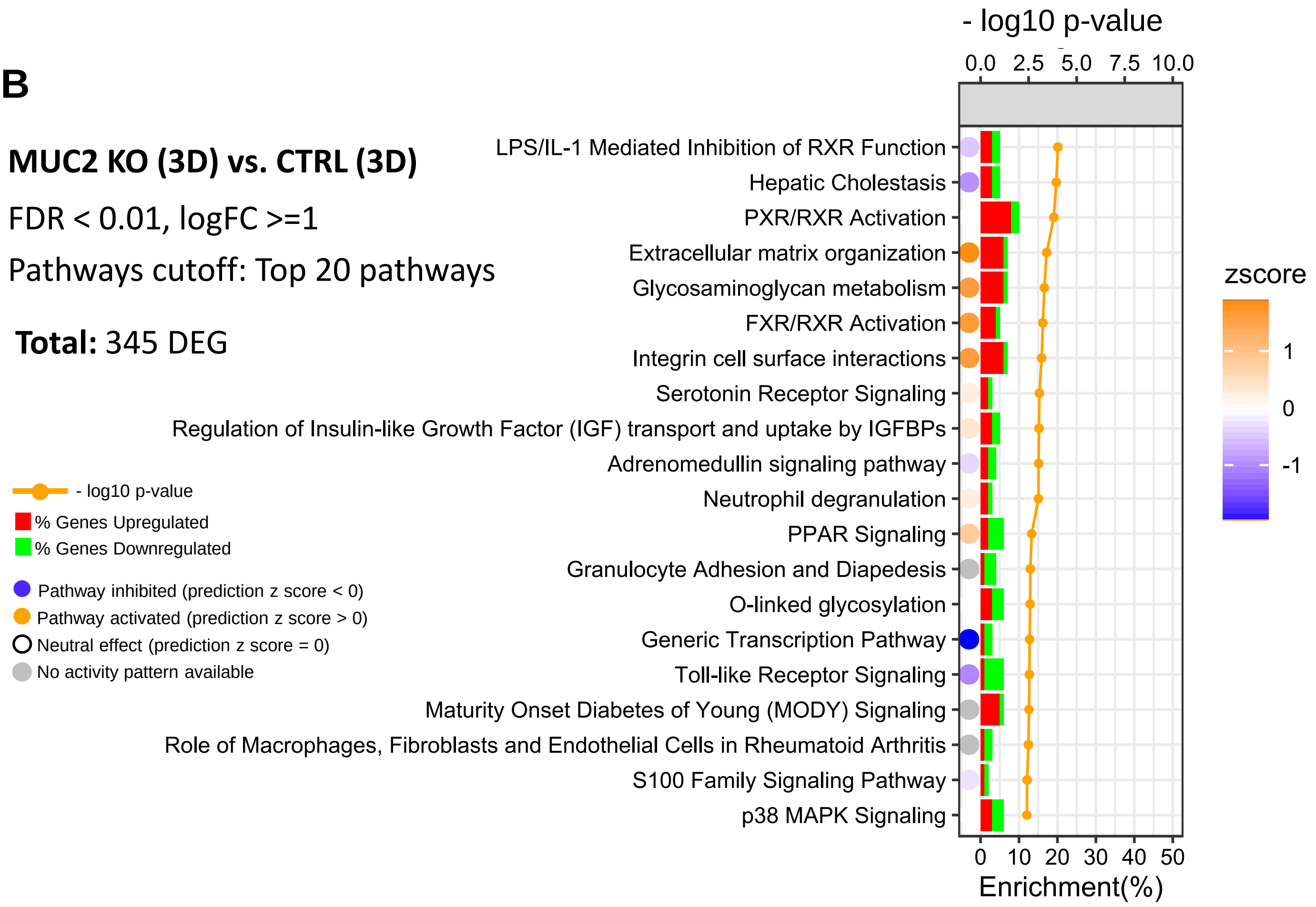

Supplementary figure 17

C

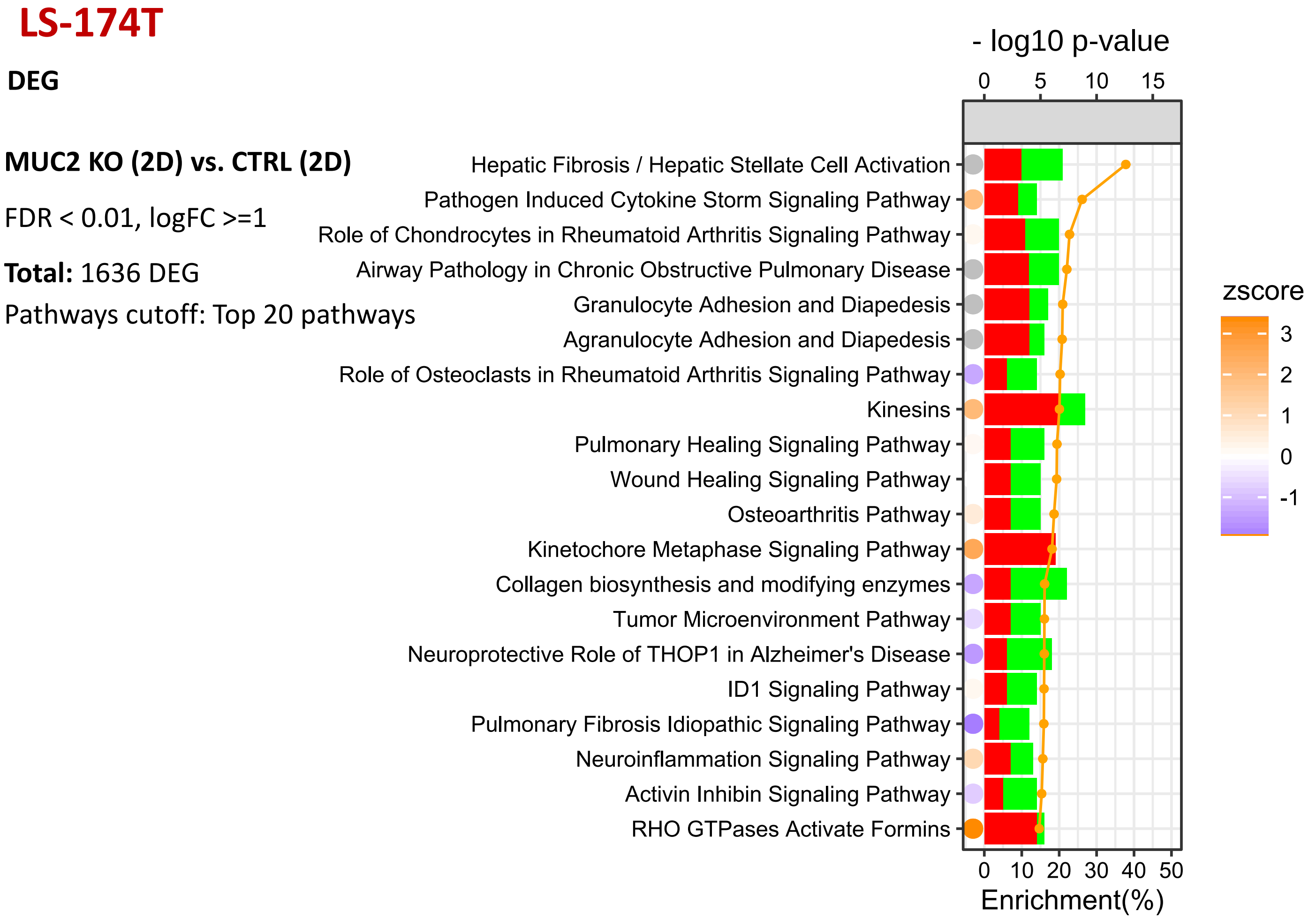

D

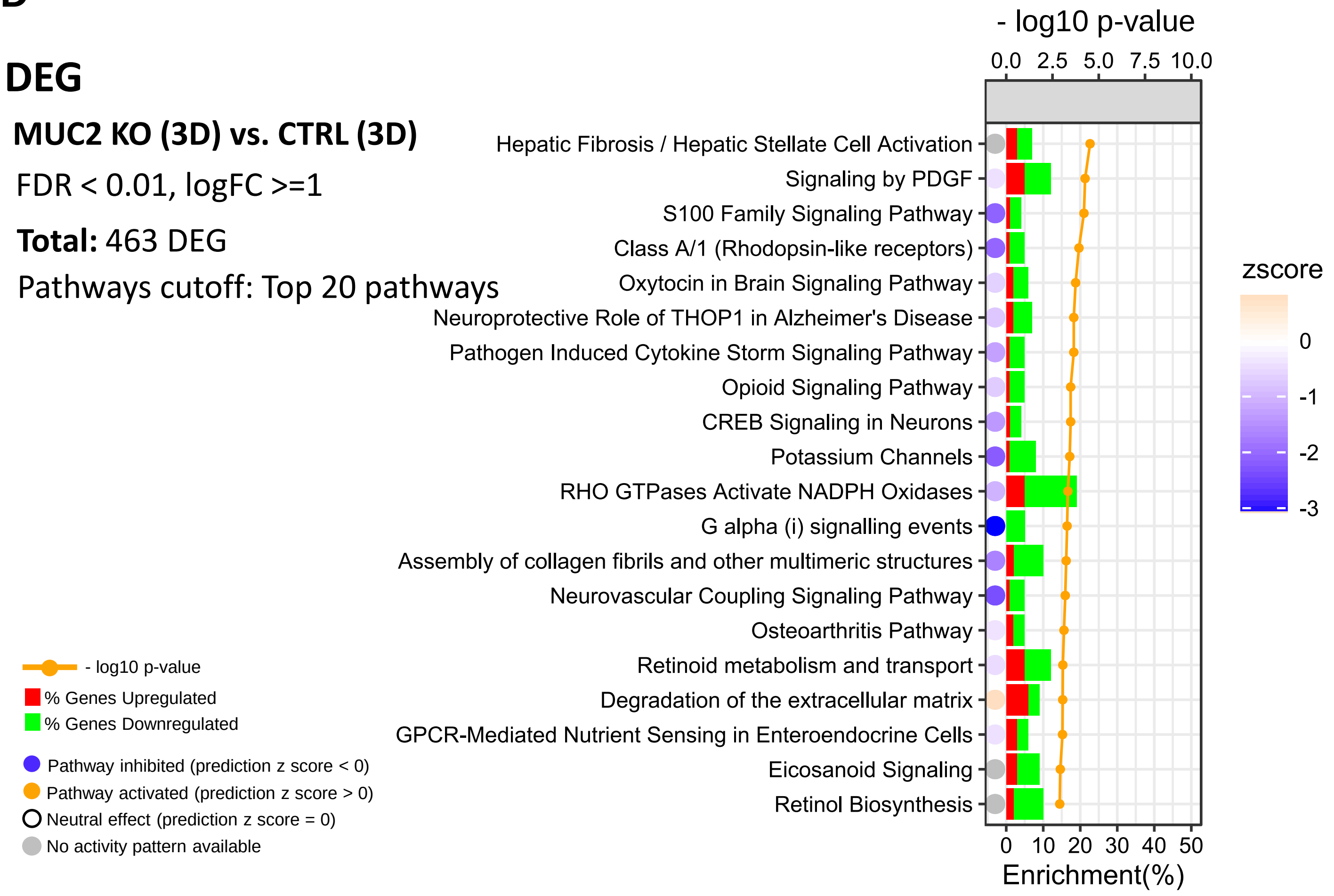

ICR score PBMCs IN

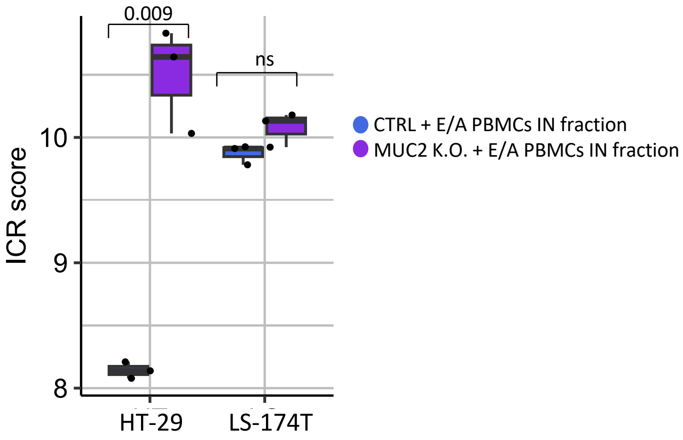

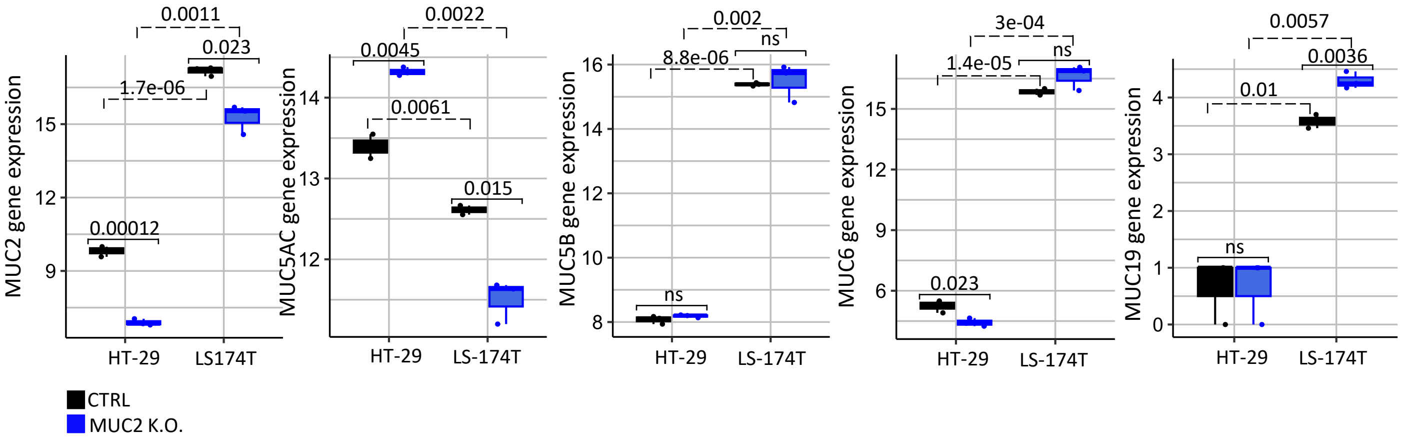
MUC2 gene expression
0.0011
0.023
1.7e-06
0.00012
15
12
9
HT-29
LS174T
MUC5AC gene expression
0.0022
0.0045
0.0061
0.015
14
13
12
HT-29
LS-174T
MUC5B gene expression
0.002
ns
8.8e-06
ns
16
14
12
10
8
HT-29
LS-174T
MUC6 gene expression
3e-04
1.4e-05
ns
0.023
15
12
9
6
HT-29
LS-174T
MUC19 gene expression
0.0057
0.0036
0.01
ns
4
3
2
1
0
HT-29
LS-174T
CTRL
MUC2 K.O.

Supplementary figure 20

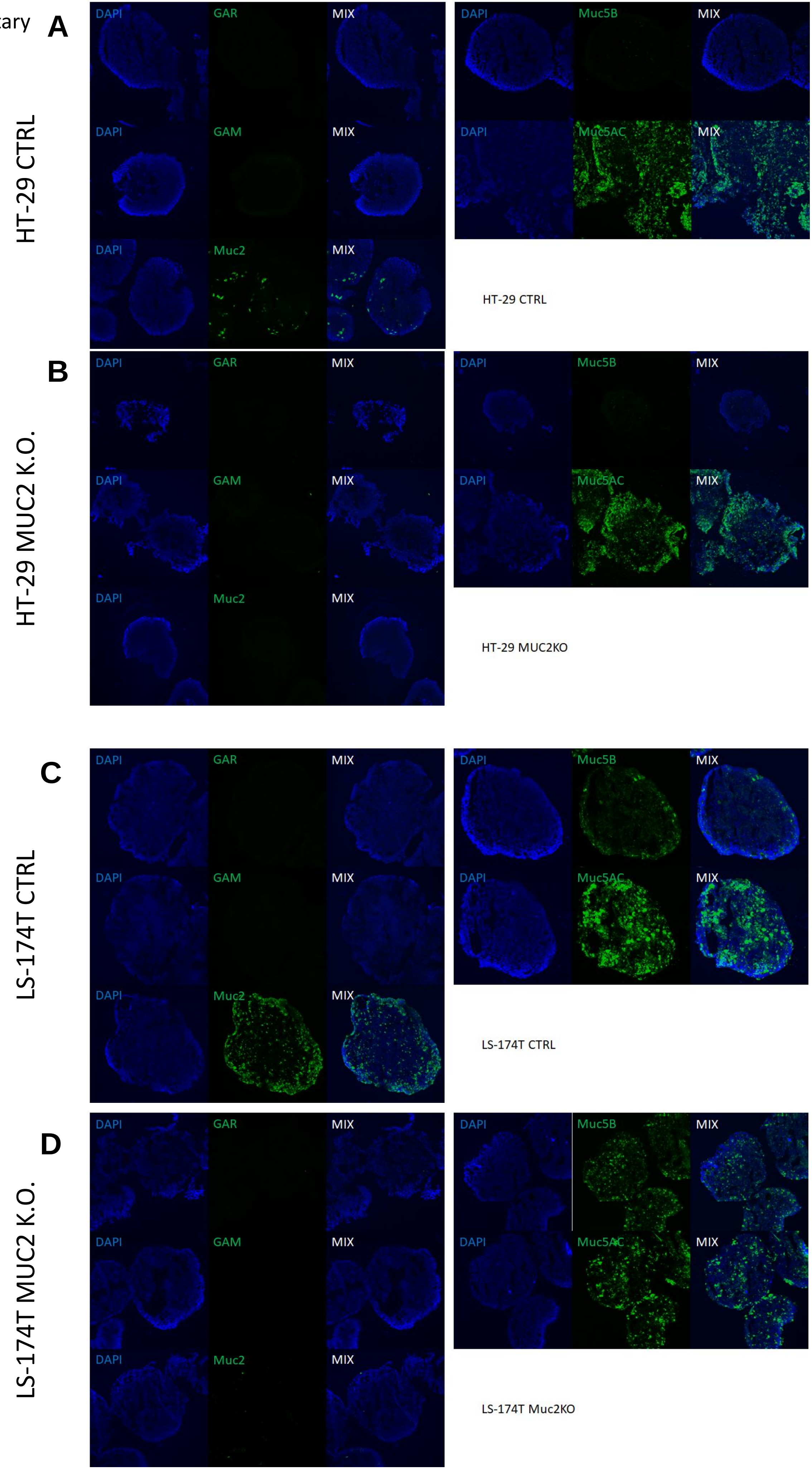

Supplementary figure 21

A

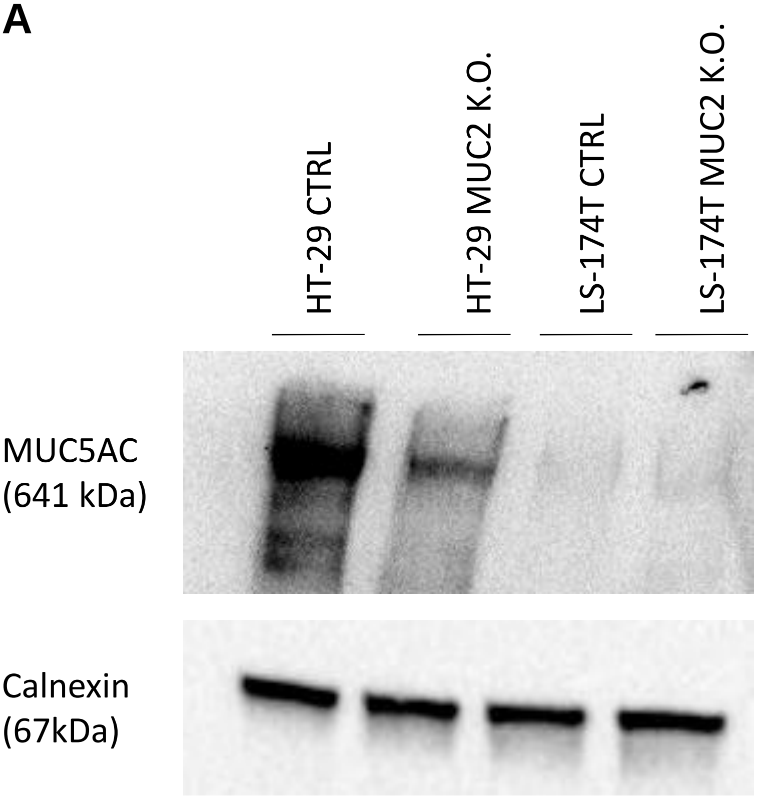

B

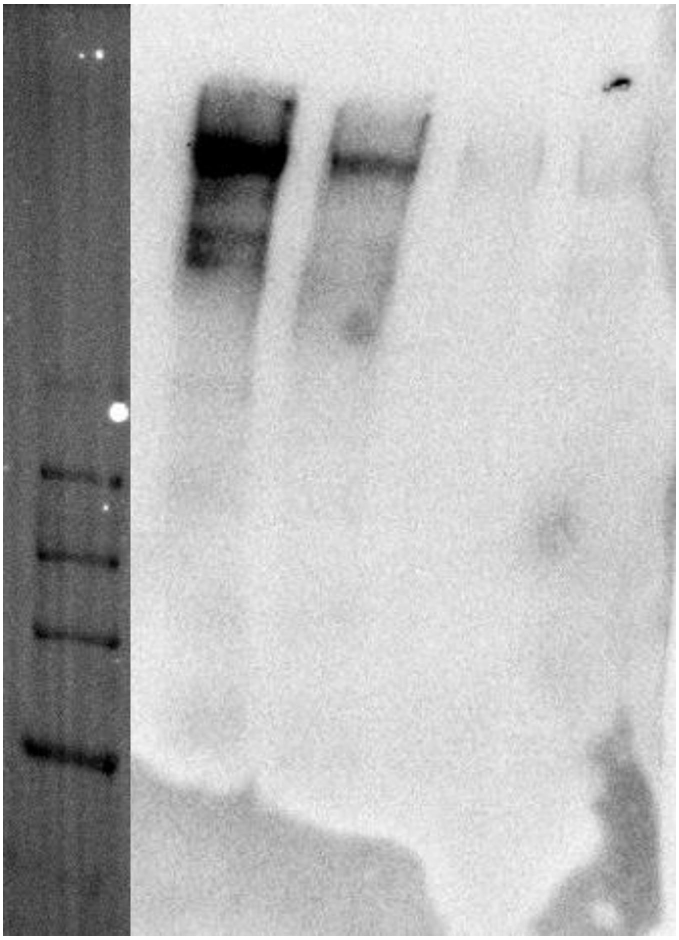

C

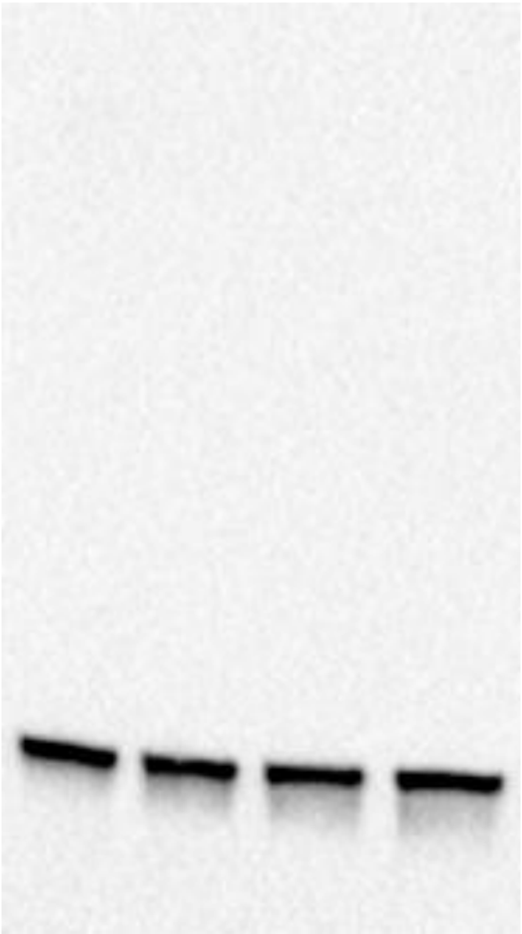

Supplement: Supplementary Figure 1 — Relative localization and orientation of the five gRNAs design for knockout of MUC2 on exon 2 and the primers used for PCR and Sanger sequencing. [file Supplementaryfile1.pdf]
